# Plant hormonal changes and differential expression profiling reveal seed dormancy removal process in double dormant plant-herbaceous peony

**DOI:** 10.1371/journal.pone.0231117

**Published:** 2020-04-02

**Authors:** Xueting Li, Riwen Fei, Zhijing Chen, Chuanzhu Fan, Xiaomei Sun

**Affiliations:** 1 Horticulture College, Shenyang Agricultural University, Shenyang, Liaoning, China; 2 Forestry College, Shenyang Agricultural University, Shenyang, Liaoning, China; 3 Department of Biological Sciences, Wayne State University, Detroit, MI, United States of America; Youngstown State University, UNITED STATES

## Abstract

Herbaceous peony (*Paeonia lactiflora* Pall.) is a popular ornamental and medicinal plant. Taking approximately six to seven months, the seeds germination under natural conditions experiences dual dormancies, which seriously affects horticultural cultivation. Few studies have been conducted on exploring both biological and molecular mechanism that regulates dormancy removal process in hypocotyls double dormant plants. Here, we first measured ABA and GA_3_ content changes at four key dormancy break stages, and then performed transcriptomic analyses to identify the differentially expressed genes (DEGs) using RNA-seq. We subsequently carried out Quantitative real-time PCR (qRT-PCR) to validate RNA-seq data. ABA content decreased during the whole dormancy removal process and GA_3_ content exhibited decreasing slightly and then increasing trend. RNA sequencing *de novo* assembly generated a total of 99,577 unigenes. 20,344 unigenes were differentially expressed in the whole dormancy release process. The qPCR results of 54 selected unigenes were consistent with the FPKM values obtained from RNA-seq. Our results summarize a valuable collection of gene expression profiles characterizing the dormancy release process. The DEGs are candidates for functional analyses of genes affecting the dormancy release, which is a precious resource for the on-going physiological and molecular investigation of seeds dormancy removal in other perennial plants.

## Introduction

*Paeonia lactiflora* (herbaceous peony), a herbaceous perennial flower in the family *Paeoniaceae*, has excellent ornamental and medicinal values. Native to central and eastern Asia, *P*. *lactiflora* has been widely grown in China for more than 3900 years. With its big flower and beautiful floral color, *P*. *lactiflora* has been primarily grown as garden plants and also cultivated as commercially cut flower. Through long-term systemic evolution subject to natural adaptation, *P*. *lactiflora* seeds adopt hypocotyl and epicotyl dormancy in the wild, which is a special dormancy characteristic in plants [1]. Generally, when the temperature declines in autumn, the hypocotyl elongates and grows out of the radicles. After the winter cold period passes, the epicotyl dormancy is broken when the temperature warms up in spring. It takes six to seven months under natural conditions for *P*. *lactiflora* seeds to break dormancy. Incomplete removal of dormancy during seed germination leads to a decrease in germination rate, which seriously affects actual cultivation and final plant and floral production. Previously, although we have analyzed two different developmental stages of peony seeds, it is not enough to better understand the whole complex double dormancy release process in herbaceous peony [2]. This study will give a comprehensive and systematic perspective on seed dormancy and germination, a key process in plant growth and development.

Dormancy is a blockage of germination. Even under favorable conditions, by intrinsic factors, dormancy is considered as a survival strategy for plants to survive seasonal harsh climatic conditions [3,4]. With mostly focused on seed dormancy and germination mechanisms, seed biology has always been one of fundamental concentrations in plant research [5]. For the reasons of numerous physiological, biochemical and molecular processes regulations, seed dormancy and germination is a complex, and sometimes long period process [6], which is controlled by a combination of genetic and external environmental factors [7–9]. Environmental conditions, including moisture, light and temperature, are external factors that affect seed dormancy and germination. Endogenous factors, e.g., plant hormones, are crucial in regulating seed dormancy and germination. Many plant hormones have been identified involving in the regulation of seed dormancy and germination. Extensive studies have shown that abscisic acid (ABA) and gibberellin acid (GA) are the primary endogenous factors that regulate the transition from dormancy to germination, and they regulate this process antagonistically [10–16]. In addition, ethylene (ETH), brassinosteroid (BR), and cytokinin (CTK) also participate in seed dormancy and germination regulation by directly or indirectly involving in ABA or GA signaling pathways [17–19].

Seed dormancy and germination studies have witnessed appreciable progresses in the last two decades. However, how those complex signal transduction and gene expression regulation pathways are regulated in a coordinated and sequential manner during seed dormancy removal process, is largely unknown [5]. To better understand the dormancy release process in *P*. *lactiflora* seeds, we measured the contents of ABA and GA_3_ to elucidate regulatory mechanisms and identify the genes underlying seed dormancy in herbaceous peony, which display dual and long dormancy process. Here, we obtained transcriptomes of four different seeds dormancy releasing stages in *P*. *lactiflora*. We *de novo* assembled and annotated unigenes based on transcripotomes without reference genome of *P*. *lactiflora*. We identified differentially expressed genes and analyzed their biological functions based on *in silico* and *in vitro* analyses. We ultimately identify handful key candidate genes regulating seed germination and dormancy. We generated basic molecular mechanisms of dual dormancy in *P*. *lactiflora*, which will provide crucial clues for improving the germination rate of seeds, shortening the breeding cycle and using molecular techniques to cultivate new varieties of *P*. *lactiflora*.

## Materials and methods

### Plant materials and samples collection

*Paeonia lactiflora* was grown at germplasm resources nursery in Shenyang Agricultural University, Liaoning Province, China. The hybrid seeds (‘Fen Yunu’×‘Fen Yulou’) were harvested annually in August. For the transcriptome sampling, seeds (harvested at the same time) at four dormancy releasing points were collected and flash frozen in liquid nitrogen and stored at −80°C for next trail.

### Measurements of ABA and GA_3_ contents

The ABA and GA_3_ contents were measured at four dormancy removal stages, T1, T2, T3 and T4. Seeds with a weight of 0.5 g were transferred to a mortar and ground into a powder with 5 mL 80% methanol. They were left to stand overnight in darkness at 4°C. The next day, the solution was shaken using a vortex with 0.1 g polyvinyl pyrrolidone for 10 min, centrifuged at 8000 × g for 10 min and the supernatant was extracted with 2.5 mL 80% methanol twice. The combined solution was then vacuum concentrated (4°C). Add 3 mL of phosphate buffer solution (0.1 mol·L^-1^) with pH = 8.0, freeze in -80°C for 30 min, centrifuge at 4°C for 15 min (8000 × g), discarding impurities. Adjust the solution’s pH to 2.5–3.0 with 2 mol·L^-1^ HCl, extract with an equal volume of ethyl acetate three times. The combined solution was then vacuum concentrated (4°C), extracted with ethyl acetate and filtered with a 0.22 μm membrane.

ABA was determined via the UPLC-MS/MS method (Waters ACQUITY UPLC H-Class &Waters Xevo TQD); UPLC: Waters ACQUITY UPLC BEH Shield RP C18 column (2.1 mm × 50 mm, 1.7 μm particle size). The column temperature was 40°C, flowrate was 0.2 mL·min^−1^and injection volume was 3μL. The solvent system consisted of acetonitrile (A) and water (B); MS: electrospray ionization (negative mode) via multiple reaction monitoring (MRM) mode. The capillary voltage was 0.8KV, cone hole voltage was 30V, solvent removal temperature was 650°C, the desolvent gas flow rate: 1000L/Hr and the cone blowback was 5 L/Hr.

Statistical analysis of ABA and GA_3_ contents data was conducted using an analysis of variance (ANOVA) procedure of SPSS 19.0 software at a significance level P < 0.05.

### RNA extraction and transcriptome sequencing

Ten frozen seeds from each stage were pooled for total RNA extraction. Total RNA was extracted from seeds collected at T1, T2, T3 and T4 respectively by using the RNAprep pure Plant Kit (Tiangen Biotech., China) and then was subsequently used in cDNA library construction and Illumina sequencing, which was completed by Beijing Novogene Bioinformatics Technology Co. Ltd on Illumina HiSeqTM2000 sequencer. After passing sample testing using oligo (dT) magnetic bead-enriched eukaryotic mRNA, the total extracted RNA was detected with the Agilent 2100 instrument (Agilent Technologies, Palo Alto, CA, USA).

### Raw sequencing data processing

The image data file obtained by sequencing was converted into raw reads using CASAVA base calling. Joint reads were removed from raw reads, and an N ratio > 2% was used to remove the low-quality reads and obtain the clean reads. All subsequent analyses were based on the clean reads.

The raw data generated in this study have been uploaded to Sequence Read Archive (https://www.ncbi.nlm.nih.gov/sra/), BioProject ID: PRJNA589141.

### *De novo* assembly and annotation

We carried out *de novo* assembly using Trinity without reference genome. First, the contigs were assembled using the overlapping area of the reads. Second, Connected to the contig assembly sequence of into the ends cannot be extended again (unigene). The unigenes were compared with the Nr, Nt, Pfam, Swiss-Prot, GO, KEGG and KOG databases to determine the direction of the unigenes. The GO functional classification of the unigenes was performed using Blast2GO [20], and the pathway analyses were performed using the KEGG annotation service [21].

### Screening of differentially expressed unigenes, GO classification, and pathway analysis

We used the FPKM method to calculate expression of the unigenes and detected differences in expression between different samples. We compared different samples using the trimmed mean of M-values to process the standardized data. Differences were analyzed using DEGseq. The screening threshold to satisfy the conditions for a unigene would be defined as a DEG was a q-value < 0.005 and |log2 fold-change| >1.

### Gene expression profiling and KEGG pathway enrichment

Gene expression trends from T1 to T4 were analyzed and clustered using the software of Short Time-series Expression Miner (STEM) [22]. Genes were clustered into 20 expression profiles. Those profiles with P < 0.01 were separately subjected to KEGG pathway enrichment, and top five enriched pathways were focused.

### Quantitative PCR analysis

54 unigenes were chosen for validation by qRT-PCR. 26 unigenes were related to hormone metabolism and signal transduction, and the other 28 unigenes were randomly selected. Expression levels were normalized against the reference gene *PlActin* (GenBank accession no. JN105299.1), which presented stable expression during the dormancy release process of *P*. *lactiflora* seed in our previous study [23]. Gene primers were designed with Primer Premier 5.0 software. Total RNA was extracted with the RNAprep pure Plant Kit (Tiangen Biotech., China) and reverse transcribed into cDNA using the PrimeScritH RT reagent kit with the gDNA Eraser (Perfect Real Time) (Takara, Japan). qRT-PCR was performed with a StepOnePlus Real Time PCR System (Life Technologies, USA). Each reaction was performed in a total reaction mixture volume 20 μl containing 2 μl cDNA, 10 μl SYBR premix Ex taqTM (Takara, Japan), 0.8 μl each of 10 mM forward and reverse primers, 6 μl RNase-free water, and 0.4μl ROX Reference Dye (Takara, Japan). The amplification program was as follows: 95°C for 30 s, 40 cycles of 95°C for 5 s, and 60°C for 30 s. Each reaction was performed in three replicates. Expression levels of candidate genes were determined using the 2^−ΔΔCt^ method. Statistical analysis of qRT-PCR data was conducted using an analysis of variance (ANOVA) procedure of SPSS 19.0 software at a significance level P < 0.05.

## Results

### ABA and GA_3_ contents during seed dormancy release process

**Fig 1:**
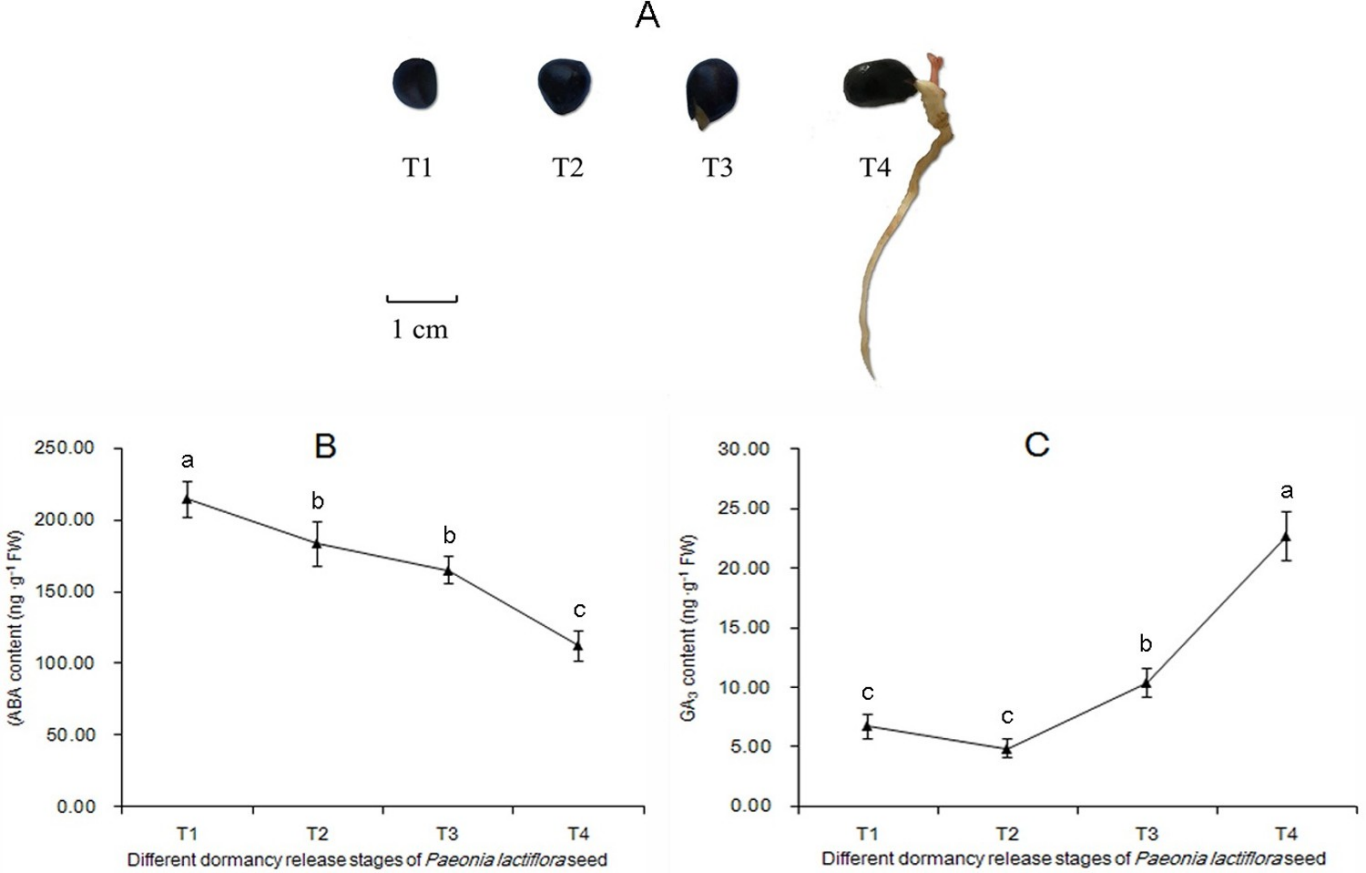
Observation and measurement of plant hormones across the dormancy release stages of herbaceous peony seeds. Seed dormancy release process (A), dry seed (T1); imbibition seed (T2); the radicle breakthrough seed coat (T3); the germ break out (T4). ABA content (B) and GA_3_ content (C), vertical bars represent standard errors of means (n = 3). Different lower case letter (a–c) indicates the significant difference among four stages at P < 0.05.

We measured the levels of ABA and GA_3_ in four consecutive points during seed dormancy release process (Fig 1A). We observed the different dynamics of ABA and GA contents during seed dormancy releasing. The ABA content gradually declined from the beginning to the end of the dormancy, exhibited insignificant change during hypocotyl dormancy removal process (from the imbibition seed T2 to the radicle breaking seed coat T3) (Fig 1B). However, GA_3_ content exhibited insignificant change during seed imbibition (from the dry seed T1 to imbibition seed T2) and then climbed through the end of dormancy (Fig 1C).

### Transcriptomic sequencing, assembly, and annotation

In total, 225,225,600 raw reads were generated from the four libraries. And the high throughput RNA sequencing obtained over 5 Gb data from each of the seed samples with Q20 all higher than 96.2% (S1 Table). After removal of adaptor sequences, ambiguous nucleotides and low-quality sequences, a total of 216,114,010 clean reads were used to assemble the transcriptome data using the Trinity method. Using overlapping information in high-quality reads, 138,473 transcripts and 99,577 assembled unigenes were generated with an average length of 767 bp and 650 bp (S2 Table). The N50 value, widely used to assess the quality of sequence assembly, was 1,281 bp and 1,044 bp, respectively. All unigenes were longer than 200 bp. The detailed length distribution of the transcripts and unigenes is shown in S1 Fig.

All assembled unigenes were aligned against the seven public databases including non-redundant protein (Nr) database, non-redundant nucleotide (Nt) database, Pfam (Protein family), Swiss-Prot, Gene ontology (GO), Kyoto Encyclopedia of Genes and Genomes (KEGG) and euKaryotic Ortholog Groups (KOG). The number and percentage of unigenes annotated by each database are summarized in S3 Table. A total of 41,401 (41.57%) unigenes were annotated in at least one databases, leaving 58,176 (58.43%) unigenes without any annotation and the low annotation rate may be attributable to the limited genomic information for *P*. *lactiflora*. Using BLASTx, we aligned 99,577 unigene sequences against the NCBI nr protein database with a cut-off E-value of 1 × 10^−5^. Among the assembled sequences, 36744 unigenes were annotated with significant BLAST results. Of these, 40.7% had very strong homology (E<1 × 10^−60^), 22.4% showed strong homology (1 × 10^−60^ < E < 1 × 10^−30^), and 37.1% showed homology (1 × 10^−30^ < E < 1 × 10^−5^) to sequences in the Nr database (S2A Fig). The similarity distribution was comparable, with 50.9% of sequences having similarities higher than 80%, and 49.1% with similarities of 18–80% (S2B Fig). With respect to species, 18.4% sequences had top matches to sequences from *Vitis vinifera*. Other frequent hits were to *Quercus suber* (6.6%), *Actinidia chinensis* var. *chinensis* (4.5%), *Juglans regia* (3.8%), *Nelumbo nucifera* (2.7%) and over 63.9% matched others (S2C Fig). GO, KEGG and KOG function classification of all the unigenes are shown in S3–S5 Figs.

### Analysis of differentially expressed genes (DEGs)

**Fig 2:**
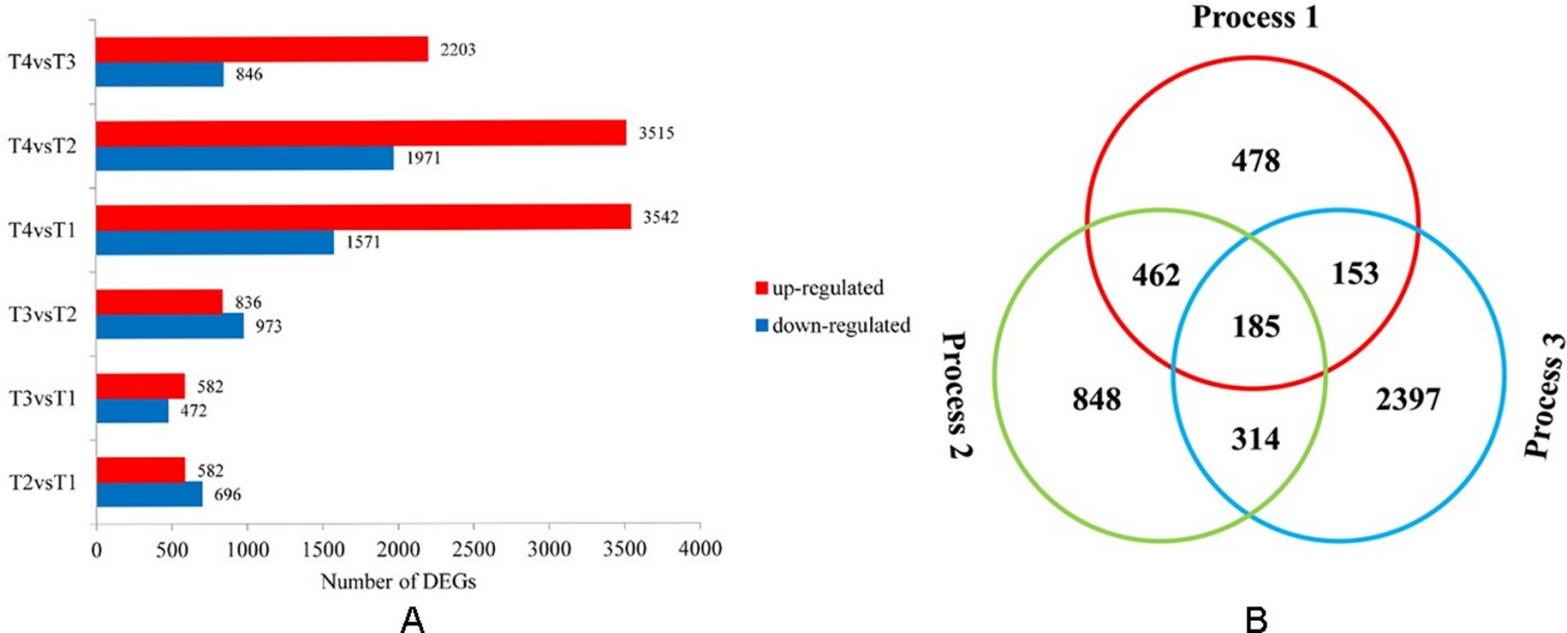
The distribution of differentially expressed genes. Numbers of differentially expressed genes involved in phase changes during seed dormancy release of herbaceous peony (A). Venn diagram analyses of differentially and stage-specific expression genes per comparison (B).

Differential expression analyses revealed changes in gene expression during different dormancy release stages and pairwise comparisons were performed between the four libraries (Fig 2A). Transition from T2 to T4 had the greatest number of differentially expressed genes (5,486) with 3,515 up-regulated and 1,971 down-regulated genes while transition from T1 to T3 had the lowest number with 582 up-regulated and 472 down-regulated. In order to better understand the key transition stages, we categorized the four stages into three processes: Process 1-seed imbibition process (from the dry seed T1 to imbibition seed T2); Process 2-hypocotyl dormancy removal process (from the imbibition seed T2 to the radicle breaking seed coat T3); Process 3-epicotyl dormancy removal process (from the radicle breaking seed coat T3 to the germ breaking seed coat T4). We compared gene expression among three processes and identified DEGs for these three processes (Fig 2B). As shown in Fig 2B, comparisons of the three processes identified 1,278, 1,809 and 3,049 DEGs in Process 1, Process 2, and Process 3, respectively. Of these DEGs, 185 were significantly regulated in the whole seed dormancy removal process. 462 genes were differentially regulated in both Process 1 and Process 2, 314 genes in both Process 2 and Process 3, 153 genes in both Process 1 and Process 3. There were 478, 848 and 2,397 genes differentially expressing in Process 1, Process 2 and Process 3, respectively (Fig 2B). The result indicated that compared with the seed imbibition process (Process 1) and the hypocotyl dormancy removal process (Process 2), the epicotyl dormancy release (Process 3) were the most complicated process given highest DEGs participated.

### GO analysis of DEGs

Pair-wise comparison of DEGs between successive stages revealed global changes in phase changes during dormancy removal process of *P*. *lactiflora* seed. Ten biological pathways were identified to be enriched based on GO analyses of the up-regulated and down-regulated genes during phase transitions.

During the seed imbibition process (Process 1), ten most significantly enriched up-regulated pathways were mainly involved in biological metabolism (e.g., ATP metabolic process, purine nucleoside triphosphate metabolic process, and other ribonucleoside or ribonucleotide processes), which indicated that in this stage seeds start to be active. We further identified enriched down-regulated GO categories to better understand seed imbibition stage. These GO terms included molecular functions and biological processes. Molecular functions were mainly about binding (nucleic acid, protein, DNA and heterocyclic compound). Biological processes were related to transcription, nucleobase-containing compound metabolic process and RNA biosynthetic process (Table 1).

**Table 1:** Changes identified by GO analysis during T1 to T2.

Change pattern	Gene ontology term	Term type	Number of DEGs	*P*-value	Corrected *P*-value
**Up regulated**	Gluconeogenesis	Biological process	8	0.000102672	0.0151219
	Hexose biosynthetic process	Biological process	8	0.000102672	0.0151219
	Monosaccharide biosynthetic process	Biological process	8	0.000102672	0.0151219
	Glucose metabolic process	Biological process	8	0.000233644	0.0151219
	ATP metabolic process	Biological process	14	0.000251262	0.0151219
	Glycolytic process	Biological process	7	0.000280245	0.0151219
	ATP generation from ADP	Biological process	7	0.000280245	0.0151219
	Purine nucleoside diphosphate metabolic process	Biological process	7	0.000280245	0.0151219
	Purine ribonucleoside diphosphate metabolic process	Biological process	7	0.000280245	0.0151219
	Ribonucleoside diphosphate metabolic process	Biological process	7	0.000280245	0.0151219
**Down regulated**	Nucleic acid binding	Molecular function	103	2.06E-09	8.32E-07
	Protein binding	Molecular function	117	7.18E-08	9.86E-06
	Binding	Molecular function	234	7.32E-08	9.86E-06
	Nucleic acid metabolic process	Biological process	98	1.15E-07	0.000106
	DNA binding	Molecular function	63	3.60E-06	0.00036333
	Transcription, DNA-templated	Biological process	58	6.42E-06	0.001628
	Nucleic acid-templated transcription	Biological process	58	6.42E-06	0.001628
	Nucleobase-containing compound metabolic process	Biological process	107	7.76E-06	0.001628
	RNA biosynthetic process	Biological process	58	8.87E-06	0.001628
	Heterocyclic compound binding	Molecular function	138	5.22E-05	0.003283574

A set of biological processes were up-regulated during Process 2, the hypocotyl dormancy release process. The up-regulated genes were significantly enriched in two main categories: biological process and molecular function. The enriched biological process mainly involved in compound metabolic process (e.g., carbohydrate, single-organism and lysine metabolic, etc.). The enriched molecular function included catalytic activity, NAD binding and hydrolase activity. The most significantly down-regulated processes were transcription factors (e.g., nucleic acid binding transcription factor activity and transcription factor activity, sequence-specific DNA binding), and biosynthetic and metabolic processes (e.g., RNA metabolic process, regulation of nitrogen compound metabolic process and some other regulation processes) (Table 2).

**Table 2:** Changes identified by GO analysis during T2 to T3.

Change pattern	Gene ontology term	Term type	Number of DEGs	*P*-value	Corrected *P*-value
**Up regulated**	Carbohydrate metabolic process	Biological process	54	4.98E-09	4.98E-06
	Single-organism metabolic process	Biological process	141	3.78E-07	0.000188794
	Catalytic activity	Molecular function	241	5.06E-07	0.000229
	NAD binding	Molecular function	15	9.46E-07	0.000229
	Hydrolase activity	Molecular function	107	2.24E-06	0.000361
	Lysine metabolic process	Biological process	11	2.49E-05	0.000188794
	Carboxylic acid metabolic process	Biological process	47	2.89E-05	0.001022335
	Small molecule metabolic process	Biological process	74	3.00E-05	0.001022335
	Aromatic amino acid family metabolic process	Biological process	14	3.06E-05	0.001022335
	Oxoacid metabolic process	Biological process	47	4.02E-05	0.001022335
**Down regulated**	Nucleic acid binding transcription factor activity	Molecular function	30	2.48E-06	0.000527885
	Transcription factor activity, sequence-specific DNA binding	Molecular function	30	2.48E-06	0.000527885
	RNA metabolic process	Biological process	83	4.54E-06	0.002386224
	Regulation of primary metabolic process	Biological process	64	6.33E-06	0.002386224
	Regulation of nitrogen compound metabolic process	Biological process	62	8.70E-06	0.002386224
	Regulation of macromolecule metabolic process	Biological process	64	8.86E-06	0.002386224
	Regulation of cellular metabolic process	Biological process	64	1.32E-05	0.002832645
	Regulation of gene expression	Biological process	57	5.55E-05	0.008608474
	Regulation of metabolic process	Biological process	64	5.99E-05	0.008608474
	Regulation of cellular macromolecule biosynthetic process	Biological process	56	7.97E-05	0.008608474

Similar to the hypocotyl dormancy removal stage, up-regulated biological pathways in the epicotyl dormancy release (Process 3) also included catalytic activity, hydrolase activity and single-organism metabolic process. Therefore, during dormancy breaking, seeds have an exuberant metabolic activity. Besides, oxidoreductase activity, lipid metabolic process and oxidation-reduction pathways also enriched. The significantly down-regulated biological pathways in epicotyl dormancy included molecular functions and biological processes (Table 3). Binding and nutrient reservoir activity were enriched in molecular function category (Table 3).

**Table 3:** Changes identified by GO analysis during T3 to T4.

Change pattern	Gene ontology term	Term type	Number of DEGs	*P*-value	Corrected *P*-value
**Up regulated**	Catalytic activity	Molecular function	593	1.50E-21	1.01E-18
	Oxidoreductase activity	Molecular function	154	1.21E-08	4.04E-06
	Lipid metabolic process	Biological process	77	1.45E-08	1.90E-05
	Oxidation-reduction process	Biological process	157	5.10E-08	2.49E-05
	Single-organism metabolic process	Biological process	316	5.69E-08	2.49E-05
	Hydrolase activity	Molecular function	225	2.88E-07	6.45E-05
	Cofactor binding	Molecular function	60	7.09E-07	0.000118886
	Coenzyme binding	Molecular function	49	6.78E-06	0.000909413
	Hydrolase activity, acting on ester bonds	Molecular function	69	8.84E-06	0.00098852
	Cofactor metabolic process	Biological process	59	5.57E-06	0.001827699
**Down regulated**	Nucleic acid binding	Molecular function	125	1.50E-15	5.82E-13
	RNA binding	Molecular function	41	1.55E-08	3.02E-06
	Binding	Molecular function	251	2.45E-08	3.17E-06
	RNA metabolic process	Biological process	85	7.90E-08	3.76E-05
	Nucleic acid metabolic process	Biological process	102	1.36E-07	4.31E-05
	RNA splicing, via transesterification reactions with bulged adenosine as nucleophile	Biological process	42	5.30E-07	0.000100908
	mRNA splicing, via spliceosome	Biological process	42	5.30E-07	0.000100908
	Heterocyclic compound binding	Molecular function	153	2.18E-06	0.00017204
	Organic cyclic compound binding	Molecular function	153	2.21E-06	0.00017204
	Nutrient reservoir activity	Molecular function	7	3.05E-06	0.000198022

### Expression profiling and KEGG pathway enrichments analysis of DEGs

Gene expression patterns throughout the seed dormancy removal process were classified into 20 profiles. STEM analysis revealed that eight profiles including 0, 6, 7, 9, 10, 12, 13 and 19 had a P-value lower than 0.01 (S6 Fig). All eight profiles showed different gene expression patterns. Five enriched KEGG pathways were listed according to the Q-value.

Profile 19 included 1,190 up-regulated unigenes during the whole dormancy release process from T1 to T4. Top five most significantly enriched pathways were related to biosynthesis (secondary metabolites and phenylpropanoid) and metabolism (metabolic pathways, glycine, serine and threonine metabolism, ascorbate and aldarate metabolism) (S4 Table).

Profile10 had 1,340 up-regulated unigenes in the epicotyl dormancy removal status (from T3 to T4). Five significantly enriched pathways included metabolic pathways, peroxisome, photosynthesis, oxidative phosphorylation, nicotinate and nicotinamide metabolism (S4 Table).

Profiles 6 included 707 down-regulated unigenes during seed imbibition process but up-regulated in hypocotyl and epicotyl dormancy release process. Significantly enriched pathways (Q-value < 0.05) included fatty acid biosynthesis and metabolism, glycosaminoglycan degradation and sphingolipid metabolism. Similar to profile 19 and profile 10, metabolic pathways was significantly enriched in profile 6 (S4 Table).

Profile 13 included 479 up-regulated unigenes in seed imbibition stage and then down-regulated during in hypocotyl and epicotyl dormancy release processes. Spliceosome and RNA transport were the most significant pathways. mRNA surveillance pathway, peroxisome, nucleotide excision repair were also involved in this profile (S4 Table).

Profile 12 included 428 lowest expressed unigenes during seed imbibition status but up-regulated during hypocotyl dormancy release process and then maintained the higher expression level in the epicotyl dormancy removal stage. Similar to profile 19, biosynthesis of secondary metabolites and metabolic pathways were significantly enriched in this profile. Two more significantly enriched pathways were starch and sucrose metabolism and carbon metabolism (Q-value < 0.05). Phenylpropanoid biosynthesis was also enriched, but it was not statistically significant (S4 Table).

Profile 7 had 414 the highest expression unigenes during seed imbibition stage, and they were down-regulated during hypocotyl dormancy release process and then had the lowest expressive levels in epicotyl dormancy removal stage. Five significantly enriched pathways (Q-value < 0.05) were mRNA surveillance pathway, spliceosome, sulfur metabolism, ribosome biogenesis in eukaryotes and isoflavonoid biosynthesis (S4 Table).

Profile 9 included 419 insignificantly different expression unigenes from T1 to T3, which was same as profile 10. However, there was a totally different expression trend from T3 to T4 comparing to profile 10. These down-regulated unigenes were significantly enriched in spliceosome pathway and ribosome biogenesis in eukaryotes. In addition, some other related pathways, but not significantly enriched, were protein processing in endoplasmic reticulum, nitrogen metabolism and carotenoid biosynthesis (S4 Table).

Profile 0 included 329 down-regulated unigenes during the whole dormancy release process from T1 to T4. Spliceosome was the only one significantly enriched pathway (Q-value < 0.05). The other four pathways, including RNA polymerase, pyrimidine metabolism, stilbenoid, diarylheptanoid and gingerol biosynthesis and nucleotide excision repair, were not statistically significant.

### Plant hormone metabolism and signal transduction

**Fig 3:**
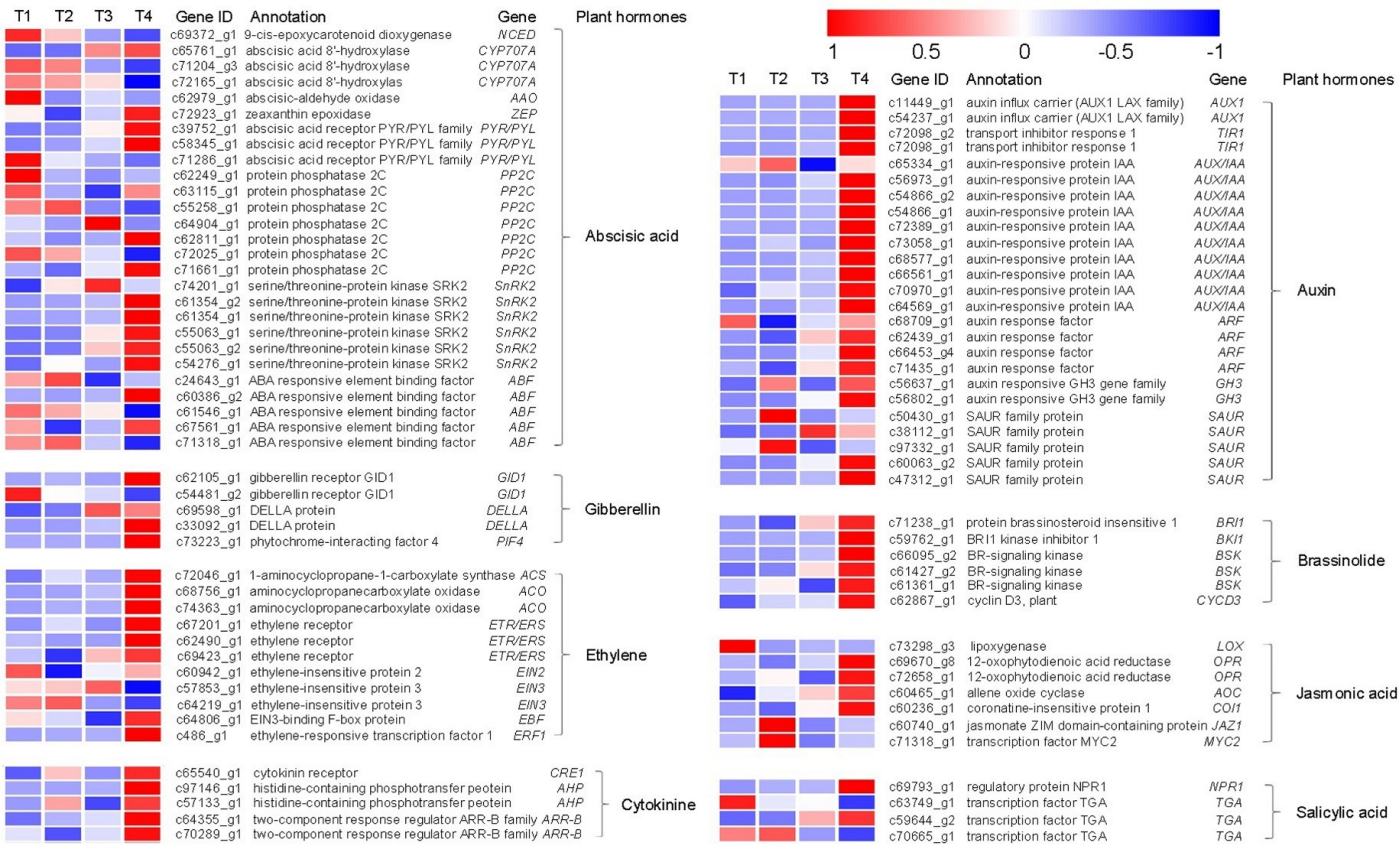
Heatmap of the expressed genes assigned to hormone mentalism and signal transduction pathway in the four transcriptomes, T1, T2, T3, T4. Dry seed (T1); imbibition seed (T2); the radicle breakthrough seed coat (T3); the germ break out (T4).

Plant hormones have been reported to play an important role in seed dormancy release. We found that many genes associated with plant hormone synthesis and degradation differentially expressed during the dormancy release of peony seeds (Fig 3). As shown in the Fig 3, among eight different plant hormones, whose metabolism or signal transduction were involved, abscisic acid, auxin, ethylene derivatives contain the most different expression. The different sequence of other five plant hormones was relatively low.

### Quantitative PCR validation of DEGs

**Fig 4:**
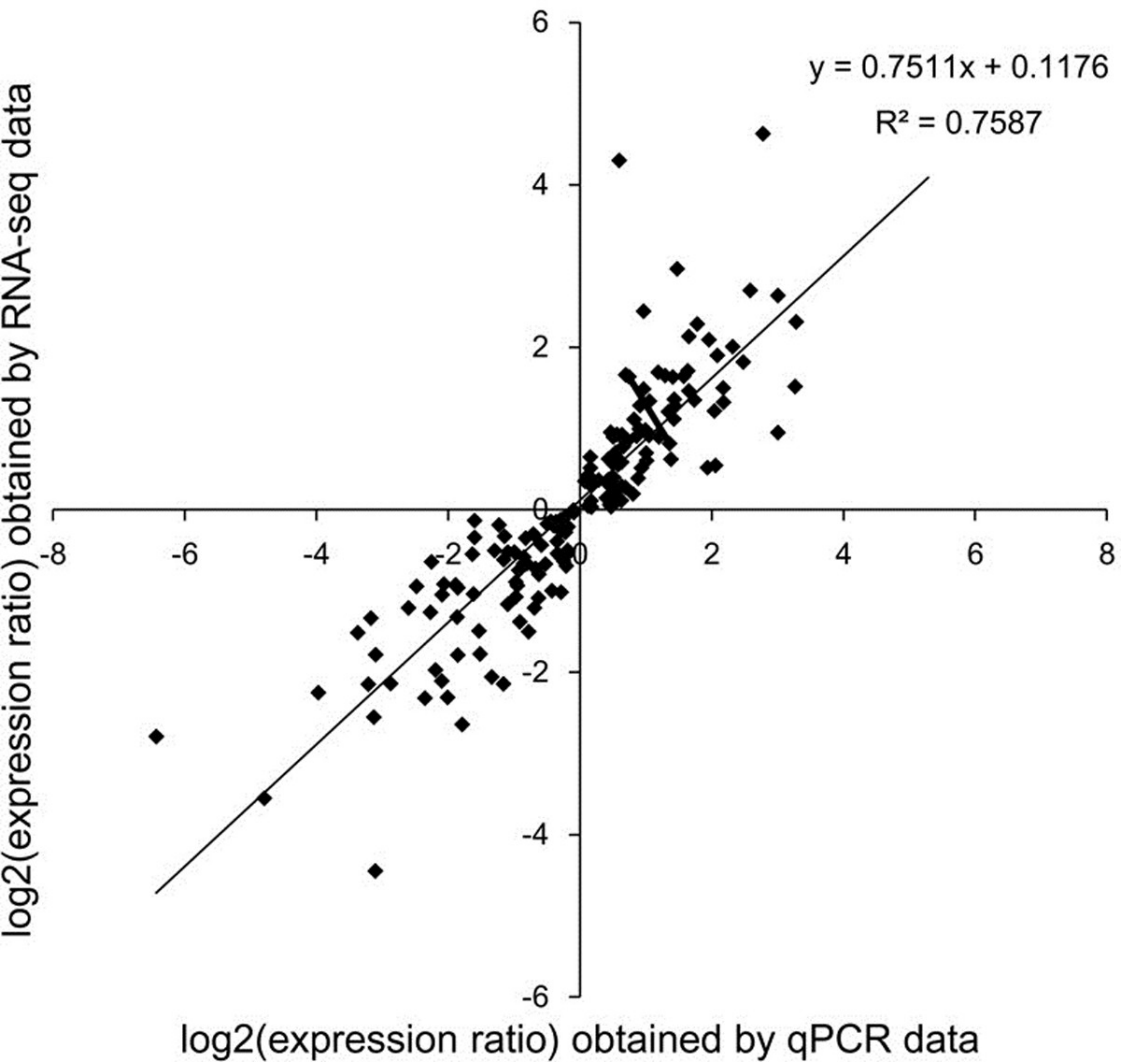
Correlation of relative gene expression levels obtained from RNA-Seq and from qPCR. The expression ratio was the ratio of gene expression level at one stage against the one at previous stage.

To validate the accuracy and reproducibility of the transcriptome analysis, gene-specific primers were designed for 54 DEGs, which have different expression patterns for qRT-PCR analysis (S7 Fig). The primers were listed in S5 Table. Scatterplots were generated by comparing the log2 fold changes determined by transcriptome analysis and qRT-PCR (Fig 4). The correlation between the RNA-seq and qRT-PCR results was evaluated by linear regression analysis, a highly significant correlation (R^2^ = 0.7587) was found between the two methods, indicating good reproducibility and validating our expression profile analysis.

## Discussion

Seed dormancy and germination are opposite concepts describing seed life activities. Seed dormancy means that intact viable seeds do not germinate temporarily under favorable conditions for germination [24]. Seed germination begins with seed imbibition, ends with the extension of the embryo shaft, usually the radicle breaking through seed coat, which is also called ‘visible germination’. This means the entire germination process is completed. However, the germination in *P*. *lactiflora* seed is special, because it has double dormancy characteristics. The dormancy removal process in *P*. *lactiflora* seed has a certain order. It must start the hypocotyl dormancy removal at first (the radicle breaking through seed coat), and then subsequently remove the epicotyl dormancy (germ breaking through seed coat). Only when the radicle is elongated to a certain extent (generally 3–4 cm based on our observation), and after a certain period of low temperature treatment, the epicotyl dormancy can be relieved [25]. If low temperature treatment is absent, only the radicle can grow but epicotyl is impossible to stick out the seed coat for the germ. There will be no seedlings. For this *P*. *lactiflora*, the seed application value of only the long radicle is very small, so its germination is different from the traditional definition of germination. From this perspective, we are more willing to call its germination as the dormancy release process.

The dormancy and germination of seeds are crucial developmental stages in plant life cycle. We investigated the complex molecular biological mechanisms underlying seed dormancy removal process in *P*. *lactiflora*. Characterizing the transcript profiles at dormancy releasing stages provides a basis for identifying candidate genes regulating the seed dormancy and germination in herbaceous peony, which could improve our overall understanding of regulatory networks involved in perennials.

### Seed imbibition process

Germination begins with seed imbibition from the perspective of seed biology. In seed imbibition process, the physical effect of water absorption is caused by the swelling force of the colloidal substance in the seed, and the basic metabolic activity of the cells in the seed is initiated and gradually turns to be strong. Our previous research showed that the water absorption process of the peony seeds lasted for 48 hours and showed a slow-fast-slow change. Previous GO analysis of DEGs showed that in this stage, the up-regulated genes were involved in the process of carbohydrate metabolism (Table 1). Up-regulated DEGs were involved in starch and sucrose metabolism, fatty acid degradation according to the KEGG analysis (S8A-2 Fig). The endosperm cavity is formed by the hydrolysis of the cell wall catalyzed by hemicellulase and β-mannan endonuclease, and the young embryos grow toward the cavity of the endosperm [26]. After the cell wall is hydrolyzed energy-rich carbohydrate hydrolysates will facilitate the growth of late embryos. GO analysis showed that during seed imbibition process, up-regulated DEGs were involved in the biological process of ATP generation from ADP (Table 1). Therefore, during the process of seed imbibition and water absorption, the metabolic activity in the cells is initiated due to the regulation of related genes, so as to provide a material basis, an energy source and other conditions for the subsequent dormancy release.

### Hypocotyl dormancy removal process

Dormancy release of the hypocotyl is a prerequisite for the germination in *P*. *lactiflora* seeds, which is completed in autumn under natural conditions [1]. The radicle extension depends on the balance between the seed coat or the endosperm’s mechanical resistance and the expansion or growth potential of the embryo volume [27]. When the seed swell is substantially complete, the water potential between the seed and the surrounding environment is close to equilibrium. In order to expand the embryo and exert pressure on the peripheral tissues, the embryonic cells must lower their own water potential to absorb more water. The degradation of storage substances (starch, fat and protein, etc.) can produce solutes (such as sugars and amino acids), which causes the cell osmotic potential to decrease, allowing the cells to continue to absorb water and expand in volume. In our study, genes related to carbohydrate metabolic process, catalytic activity and hydrolase activity increased in this process (Table 2). During the dormancy release of the hypocotyls in *P*. *lactiflora* seeds, it was found that the genes related to starch and sucrose metabolism and fat degradation were up-regulated, which was beneficial to the hypocotyl dormancy release and the radicle breaking through the seed coat (S8B-2 Fig).

### Epicotyl dormancy removal process

The shoots of seeds with epicotyl dormancy are sensitive to cold stratification only after the root has elongated [3]. With the release of hypocotyl dormancy, after the radicle protrudes from the seed coat and reaches a certain length, the peony seed needs to pass a period of low temperature to release the epicotyl dormancy, and then sprouts. No low temperature, the root will grow and produce lateral roots, but the shoot will not grow [1]. Even if a certain low temperature treatment is required to break the epicotyl dormancy, it must be grown after the root has grown to a certain length. The epicotyl dormancy of the genus *Paeonia* requires low temperature treatment, but requires the roots reach different lengths. The suitable root length for breaking epicotyl dormancy varies among species of peonies, generally from 2 cm to 6 cm [1]. For our peony hybrid seeds, it is effective to perform low temperature treatment when the root length is 3–4 cm. The underlying reason for root length affecting epicotyl dormancy release is the difference in the GA_3_/ABA ratio in the epicotyl of radicle-emerged seeds, which is mainly the result of the difference in ABA accumulation before cold stratification.

Combined with the transcriptome data, we showed that the number of DEGs in the process of dormancy release of the epicotyl is higher than those in the seed swelling and hypocotyl dormancy release process, indicating that epicotyl removal process may be more complicated (Fig 2). Through GO analysis (Table 3), KEGG analysis (S8C Fig) and STEM analysis (S4 Table), it can be known that the genes differentially expressed in this process are mainly involved in catalytic activity, oxidoreductase activity, oxidation-reduction process, lipid metabolic process, fatty acid degradation, glutathione metabolism, glyoxylate and dicarboxylate metabolism, phenylpropanoid biosynthesis, carbon metabolism, carbon fixation in photosynthetic organisms, chemical carcinogenesis, nicotinate and nicotinamide metabolism.

Regarding the complex process of epicotyl dormancy release, whether it is from the physiological and biochemical level or the molecular mechanism, we need to further study and explain. The low temperature environmental signal and the radicle length, which affect the hypocotyl dormancy release factors, are played by specific ways. It is worthwhile to design a more detailed and reasonable scientific experiment to explore. The future conclusion of this aspect will make a great contribution to the theory of seed dormancy and germination.

### Role of plant hormones in *Paeonia lactiflora* seeds dormancy release process

The decrease in ABA content was observed during seed dormancy in *P*. *lactiflora* (Fig 1B). The synthesis of ABA is controlled by a series of enzymatic catalysis reactions. 9-cis-epoxycarotenoid dioxygenase (NCED) catalyzes the cleavage reaction of epoxy-carotenoids to produce xanthoxin [28]. Abscisic aldehyde oxidase (AAO) catalyzes the last step of ABA biosynthesis, converting abscisic aldehyde to ABA [29]. In this study, ABA biosynthesis genes *NCED* (unigene c69372_g1) and *AAO* (unigene c62979_g1) had a decrease expression patterns, consistent with the content changes of ABA during seed dormancy release process (Fig 3). However, it is also known that endogenous abscisic acid levels in seeds is not only regulated by the biosynthesis, but also related to catabolism. Researches have described that the predominant pathway of ABA catabolism is via 8’-hydroxylase. *CYP707As* encode abscisic acid 8’-hydroxylase, a key enzyme in the oxidative catabolism of abscisic acid. In this study, three *CYP707As* were identified in *P*. *lactiflora* seeds. Surprisingly, they have different expression patterns in dormancy release process (Fig 3). Unigene c66761_g1 increased in the whole dormancy release process, while unigene c71204_g3 and unigene c72165_g1 had decrease trend. Previous studies have identified that four members in *Arabidopsis thaliana CYP707A* gene family (*CYP707A1* to *CYP707A4*) encode ABA 8’-hydroxylases [30]. Each *CYP707A* gene plays a distinct role during seed development and post germination growth for different regulatory mechanisms [31,32]. *CYP707A* gene members showed different expression patterns during seed stratification in *Prunus persica* to break dormancy, indicating that *PpCYP707As* plays a cross role to control the process of seed dormancy release [33]. The results in this study also indicated distinct functions and regulatory mechanisms of *CYP707As*, which await further clarification. Moreover, many genes associated with ABA signal transduction also had differential expression, suggesting that ABA does play an important role in the dormancy release of *P*. *lactiflora* seeds.

Gibberellin (GA) is an important plant hormone to promote seed germination, which has opposite actions to ABA. GA has many chemical structures, and they can determine whether it is active. GA_3_ is an important active gibberellin. Our data showed an insignificant change in GA_3_ content during seed imbibition process, and GA_3_ levels increased during hypocotyl and epicotyl dormancy release process (Fig 1C). Endogenous gibberellin promotes dormancy release in *P*. *lactiflora* seeds. Some key genes in GA signaling pathway can affect seed germination. In this study, the important components of GA signaling transduction pathway had differential expression, such as the receptor proteins GID1 (unigene c62105_g1 and unigene c54481_g2) and DELLA protein (unigene c69598_g1 and unigene c33092_g1). Studies have shown that DELLA protein is a key negative regulator of GA signaling pathway, and the dormancy DELLA protein-deficient mutant *slr1* is weakened in rice [34]. GAI, RGA and RGL are members of the DELLA superfamily. In Arabidopsis, RGL1 or RGL2 is an inhibitor of seed germination, and RGL1 regulates seed germination from GA. In the absence of exogenous GA, the RGL2 loss-of-function mutant also restored the phenotype in which the mutant could not germinate. The transcriptional expression level of RGL2 rapidly increased during seed germination and rapidly disappeared in the seed after germination [35]. However, the DELLA protein genes are basically on the rise during the whole dormancy release process, contrary to its traditional function of negatively regulating the germination. The possible reason is that it has more complex mechanism during the dormancy release in *P*. *lactiflora* seeds. That is what we need to pay attention to and give a scientific and systematic explanation in the future.

Ethylene (ETH) has a positive regulation effect on the dormancy release of seeds. The germination ability of seeds is related to ethylene level, so ethylene is involved in the regulation of seed germination and dormancy [36,37]. ETH can negatively regulate seed dormancy by antagonizing ABA and promote seed germination [8,32]. In this study, the genes involved in ethylene synthesis (*ACS*, *ACO*) had a significant upward trend in the dormancy release of the epicotyl, and the same trend also occurred in ETH receptor (*ETR/ERS*), suggesting that ethylene may be on the embryo. Axillary germination has a more pronounced promoting effect. ERFs may also play a key role in ethylene response and regulation of seed germination. The expression of *ERF1* in the dormant beech nut embryos is small, but the expression of the *ERF1* can be increased by releasing the dormant wet and cold environment. The expression of ERF1 in sunflower non-dormant embryos was 5 times higher than that in dormant embryos [38]. The transcript abundance of *ERF2* in germinated tomato seeds was higher than that of ungerminated seeds, and overexpression in transgenic plants resulted in germination of immature seeds [39]. The expression of *ERF* in the dormant release of *P*. *lactiflora* seeds showed an upward trend, which also means that *ERF* plays a positive role in this process.

Cytokinin (CTK) is also able to promote the release of dormancy in many plant seeds by interacting with other hormones. CTK promotes the release of dormancy caused by heat inhibition by increasing the biosynthesis of ethylene [40–42]. CTK can antagonize ABA to promote seed germination [43,44]. Our transcriptome data showed differential expression of three key components (*CRE1*, *AHP* and *ARR-B*) of the CTK signal transduction pathway, and the functional mechanisms of these genes are also required for subsequent exploration.

Auxin is the second hormone that can promote seed dormancy besides ABA [45]. On the one hand, auxin content affects seed dormancy. In addition, the auxin signaling transduction intensity changes in the seed, and its dormancy level is affected. Studies have found that during the process of auxin regulating seed dormancy, the auxin transcription factor (ARF) was more effective than the receptor TIR1 and the transcriptional repressor AUX/IAA [46–48], which is a key factor in the positive regulation of seed dormancy. However, Li et al. believed that auxin couldn’t directly affect seed dormancy, but mainly regulated the growth rate of radicle [49].Most genes in the SAUR(small auxin up RNA)gene family are most sensitive to auxin [50]. Overexpressed SAUR63 in *Arabidopsis thaliana* found that the hypocotyls of transgenic plants were longer, and the auxin transport content increased gradually in hypocotyls, while the axis showed a shorter trend in nine transgenic plants knocked out of the SAURs subfamily [51]. Hou et al. found a phenotype of hypocotyl growth and axonal expansion in overexpressing *AtSAUR41* plants, and auxin transport levels showed an increasing trend [52]. Therefore, auxin regulating seed hypocotyl growth and development may be mainly achieved by SAUR. Our data showed that SAURs are differentially expressed during the dormancy release of peony seeds. The hypocotyl dormancy release is an important step in *P*. *lactiflora* seeds. Five identified SUARs had different expression patterns. During the hypocotyl dormancy release, unigene c38112_g1 has a significant upward expression trend, which may play an important role in the dormancy release of *P*. *lactiflora* seeds. Unigene c50430_g1 and unigene c97332_g1 were up-regulated during seed swelling. Unigene c60063_g2 and unigene c47312_g1 were up-regulated during epicotyl dormancy release, which also means a certain effect. The specific regulatory mechanisms and processes of these genes are also well worth studying.

Brassinolide is another class of plant hormones that promote seed germination [53]. Although BR biosynthesis and signaling mutants could also germinate well, but for ABA inhibition, these mutants were more sensitive than wild type, indicating that these mutants reduce their own germination potential [54]. In Arabidopsis, the application of exogenous brassinolide could overcome the non-germination caused by GA synthesis mutations and *sleepy1* GA signal mutations [54]. Our transcriptome data showed differential expression of the genes involved in the signal transduction of brassinosteroids, indicating that brassinolide was involved in the dormancy of the peony seeds by a certain route.

Jasmonic acid and salicylic acid are also important plant hormones. At present, studies on the regulation of seed dormancy germination by jasmonic acid and salicylic acid have not been reported. Our transcriptome data showed that the synthetic genes related to jasmonic acid and the key factors of signal transduction during the dormancy release of peony seeds were differentially expressed, and so as to salicylic acid, which means that during the dormancy release of peony seeds, there may be more complex hormonal regulation networks.

## Supporting information

S1 Table Summary of sequences analysis. (DOC)

S2 Table Results of *de novo* assembly. (DOC)

S3 Table BLAST analysis of non-redundant unigenes against public databases. (DOC)

S4 Table The 8 significant expression profiles and their top 5 most significantly enriched functional pathways. (DOC)

S5 Table List of qRT-PCR primers used in this study. (DOC)

S1 Fig Length distribution of assembled transcripts and unigenes. (TIF)

S2 Fig Characteristics of homology search of *P*. *lactiflora* unigenes against the nr database. E-value distribution of the top BLAST hits for each unique sequence (A), Similarity distribution of the top BLAST hits for each unique sequence (B), Species distribution of the top BLAST hits for all homologous sequences (C).
(TIF)

S3 Fig Gene ontology classification of assembled unigenes. The 15,673 matched unigenes were classified into 3 functional categories: molecular function, biological process and cellular component.
(TIF)

S4 Fig KEGG classification of assembled unigenes. A total of 14,581 were assigned to 5 KEGG biochemical pathways: metabolism, genetic information processing, organism system, cellular processes and environmental information processing.
(TIF)

S5 Fig KOG functional classification of all unigenes sequences. 9114 unigenes showed significant similarity to sequences in KOG databases, and were clustered into 26 categories.
(TIF)

S6 Fig Profiles p-value results of the STEM analysis. DEGs were clustered into 20 expression profiles. Profiles with P < 0.01 were separately subjected to KEGG pathway enrichment.
(TIF)

S7 Fig Comparison of expression levels of 54 genes obtained by qRT-PCR analysis (left) and by RNA-seq (FPKM values) (right). Data from qRT-PCR were presented as means with standard errors of three replications. Different lower case letter (a–d) indicates the significant difference among four stages at P < 0.05, dry seed (T1); imbibition seed (T2); the radicle breakthrough seed coat (T3); the germ break out (T4).
(TIF)

S8 Fig Scatterplot of DEGs KEGG pathway enrichment from T1 to T4 by comparison. T2vsT1: total DEGs (A-1), up-regulated DEGs (A-2), down-regulated DEGs (A-3); T3vsT2: total DEGs (B-1), up-regulated DEGs (B-2), down-regulated DEGs (B-3); T4vsT3: total DEGs (C-1), up-regulated DEGs (C-2), down-regulated DEGs (C-3).
(TIF)
