# Supplementary material for: Plant hormonal changes and differential expression profiling reveal seed dormancy removal process in double dormant plant-herbaceous peony
Source: PLoS One. 2020 Apr 2;15(4):e0231117. doi: 10.1371/journal.pone.0231117 (PMC7117732; doi:10.1371/journal.pone.0231117)
Supplement: S1 Table — (DOC) [file pone.0231117.s001.doc]

**Table S1.** Summary of sequences analysis

| Sample | Raw Reads | Clean reads | Clean bases | Error(%) | Q20(%) | Q30(%) | GC(%) |
| --- | --- | --- | --- | --- | --- | --- | --- |
| T1 | 52298458 | 51245330 | 5.12G | 0.02 | 96.73 | 93.25 | 44.44 |
| T2 | 52745164 | 51926368 | 5.19G | 0.02 | 96.60 | 93.03 | 45.97 |
| T3 | 62074094 | 59583432 | 5.96G | 0.05 | 96.24 | 87.70 | 46.03 |
| T4 | 58107884 | 53358880 | 5.34G | 0.05 | 96.22 | 87.73 | 45.44 |
| Total | 225225600 | 216114010 | 21.61G |  |  |  |  |
