# Supplementary material for: Plant hormonal changes and differential expression profiling reveal seed dormancy removal process in double dormant plant-herbaceous peony
Source: PLoS One. 2020 Apr 2;15(4):e0231117. doi: 10.1371/journal.pone.0231117 (PMC7117732; doi:10.1371/journal.pone.0231117)
Supplement: S2 Table — (DOC) [file pone.0231117.s002.doc]

**Table S2.** Results of *de novo* assembly

|  | Total Number | Min Length | Mean Length | Median Length | Max Length | N50 | N90 |
| --- | --- | --- | --- | --- | --- | --- | --- |
| Transcripts | 138473 | 201 | 767 | 428 | 16796 | 1281 | 297 |
| Unigenes | 99577 | 201 | 650 | 358 | 16796 | 1044 | 263 |
