# Supplementary material for: Plant hormonal changes and differential expression profiling reveal seed dormancy removal process in double dormant plant-herbaceous peony
Source: PLoS One. 2020 Apr 2;15(4):e0231117. doi: 10.1371/journal.pone.0231117 (PMC7117732; doi:10.1371/journal.pone.0231117)
Supplement: S3 Table — (DOC) [file pone.0231117.s003.doc]

**Table S3.** BLAST analysis of non-redundant unigenes against public databases

|  | Number of unigenes | Percentage (%) |
| --- | --- | --- |
| Annotated in NR | 36744 | 36.90 |
| Annotated in NT | 24425 | 24.52 |
| Annotated in KO | 14581 | 14.64 |
| Annotated in SwissProt | 27828 | 27.94 |
| Annotated in PFAM | 15673 | 15.73 |
| Annotated in GO | 15673 | 15.73 |
| Annotated in KOG | 9114 | 9.15 |
| Annotated in all Databases | 3180 | 3.19 |
| Annotated in at least one Database | 41401 | 41.57 |
| Total Unigenes | 99577 | — |
