# Supplementary material for: Plant hormonal changes and differential expression profiling reveal seed dormancy removal process in double dormant plant-herbaceous peony
Source: PLoS One. 2020 Apr 2;15(4):e0231117. doi: 10.1371/journal.pone.0231117 (PMC7117732; doi:10.1371/journal.pone.0231117)
Supplement: S4 Table — (DOC) [file pone.0231117.s004.doc]

**Table S4.** The 8 significant expression profiles and their top 5 most significantly enriched functional pathways.

| Profile | Pathways | # of involved unigenes | Q-value |
| --- | --- | --- | --- |
| profile 19  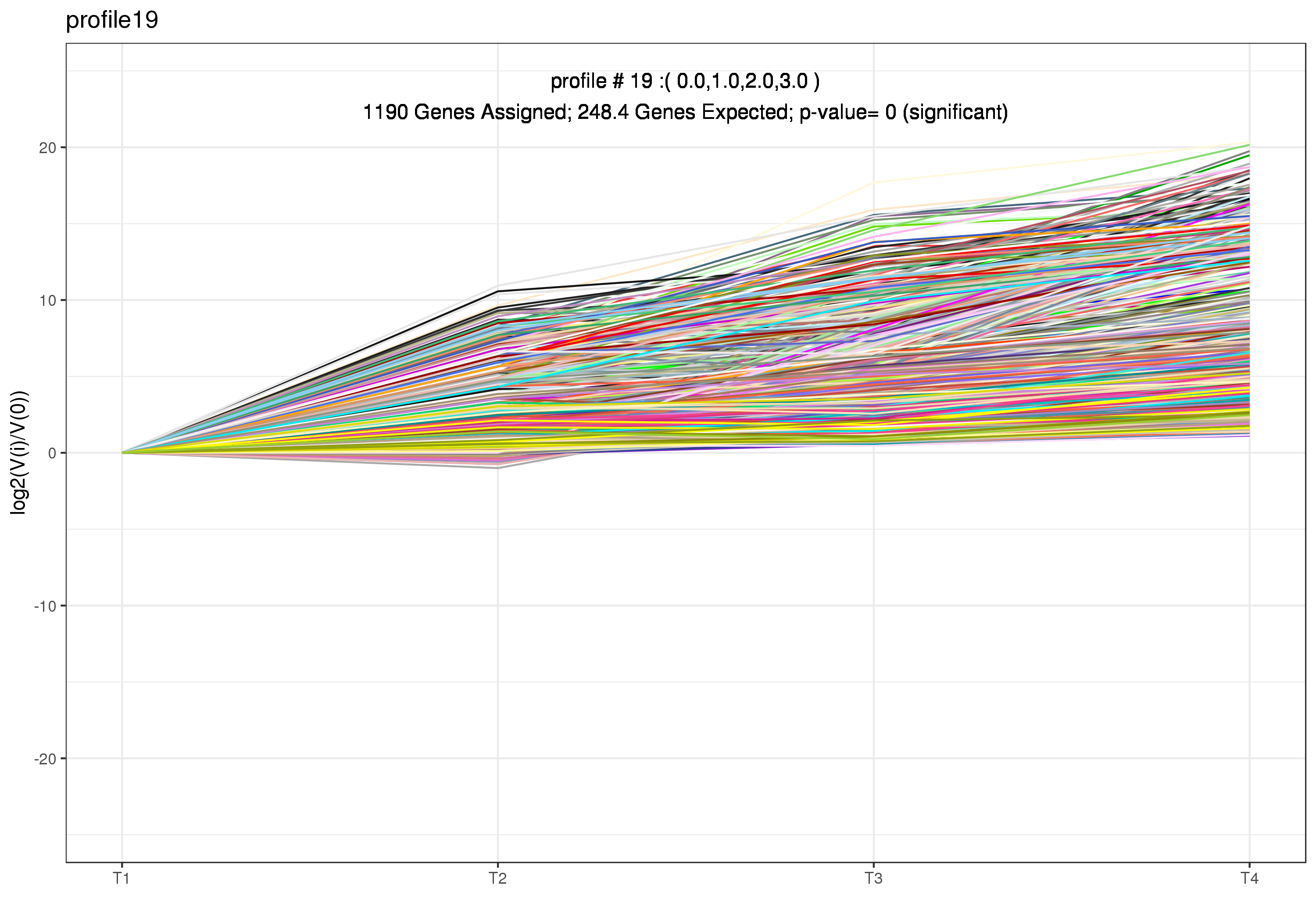 | Metabolic pathways  Biosynthesis of secondary metabolites  Glycine, serine and threonine metabolism  Ascorbate and aldarate metabolism  Phenylpropanoid biosynthesis | 160  105  11  9  18 | 3.54E-06  3.54E-06  4.27E-02  4.27E-02  4.27E-02 |
| profile 10  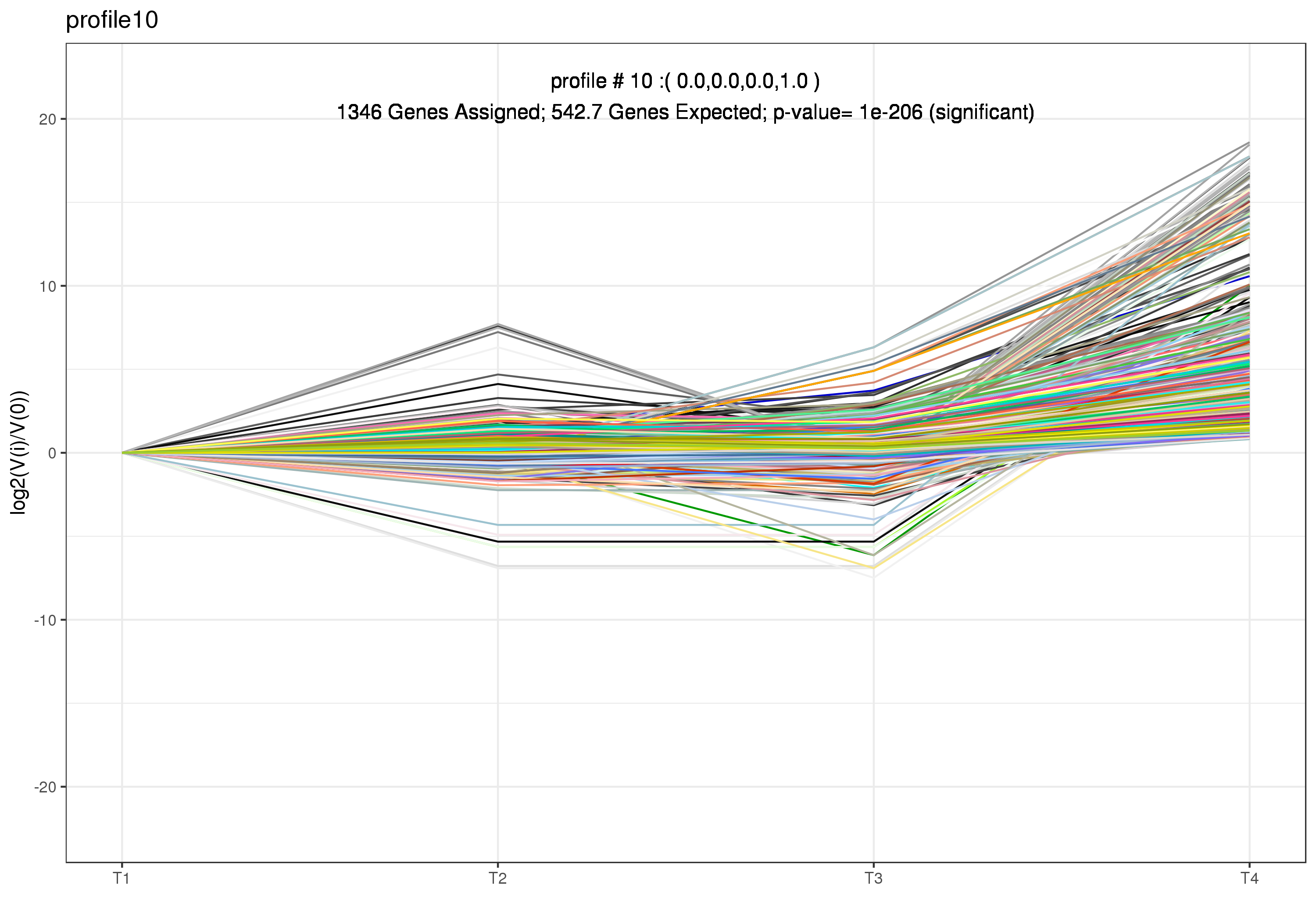 | Metabolic pathways  Peroxisome  Photosynthesis  Oxidative phosphorylation  Nicotinate and nicotinamide metabolism | 235  19  19  32  7 | 3.97E-11  3.81E-04  3.81E-04  4.82E-04  2.04E-03 |
| profile 6  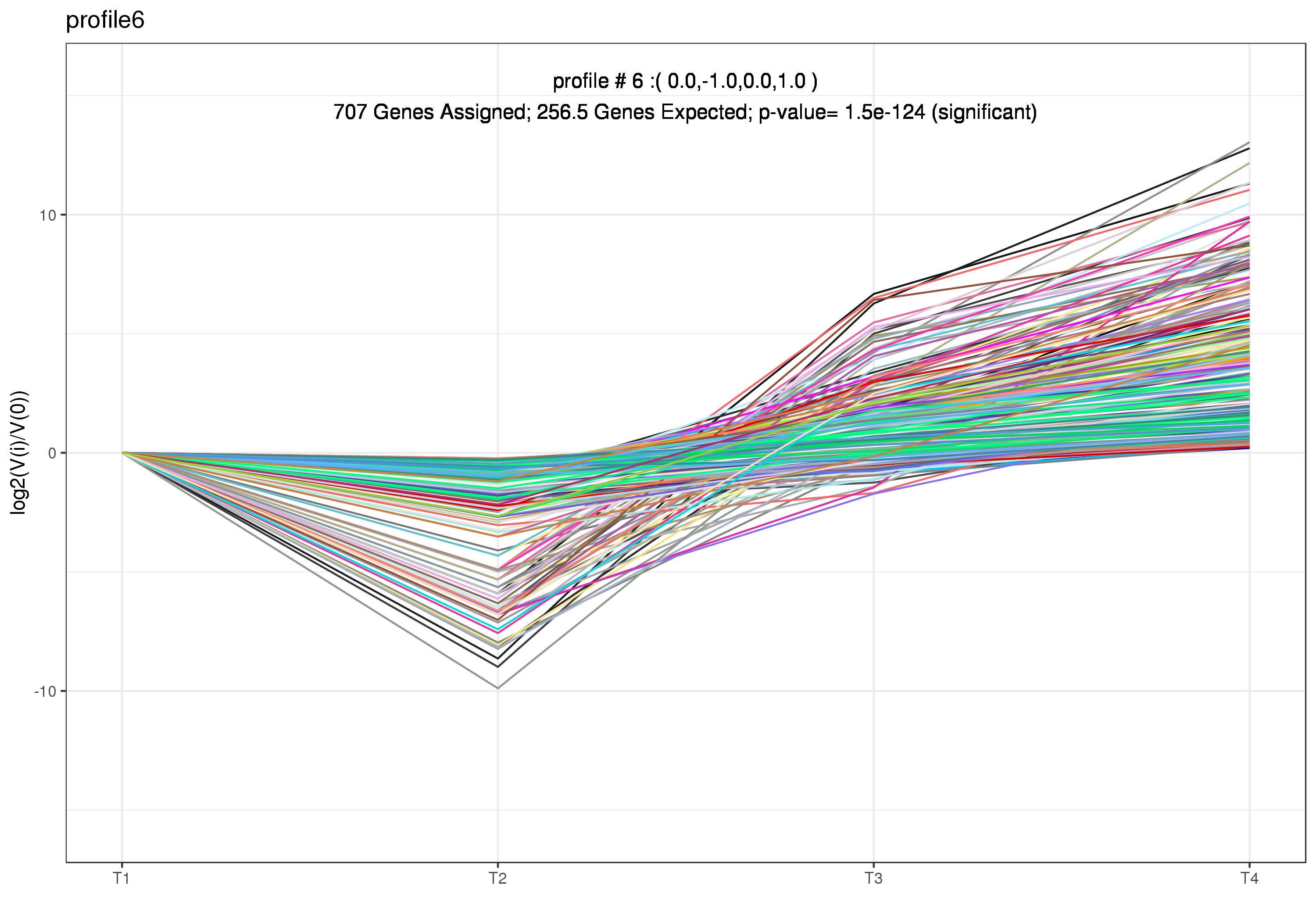 | Metabolic pathways  Fatty acid biosynthesis  Glycosaminoglycan degradation  Sphingolipid metabolism  Fatty acid metabolism | 115  8  4  6  10 | 1.19E-06  9.48E-03  1.13E-02  1.30E-02  1.30E-02 |
| profile 13  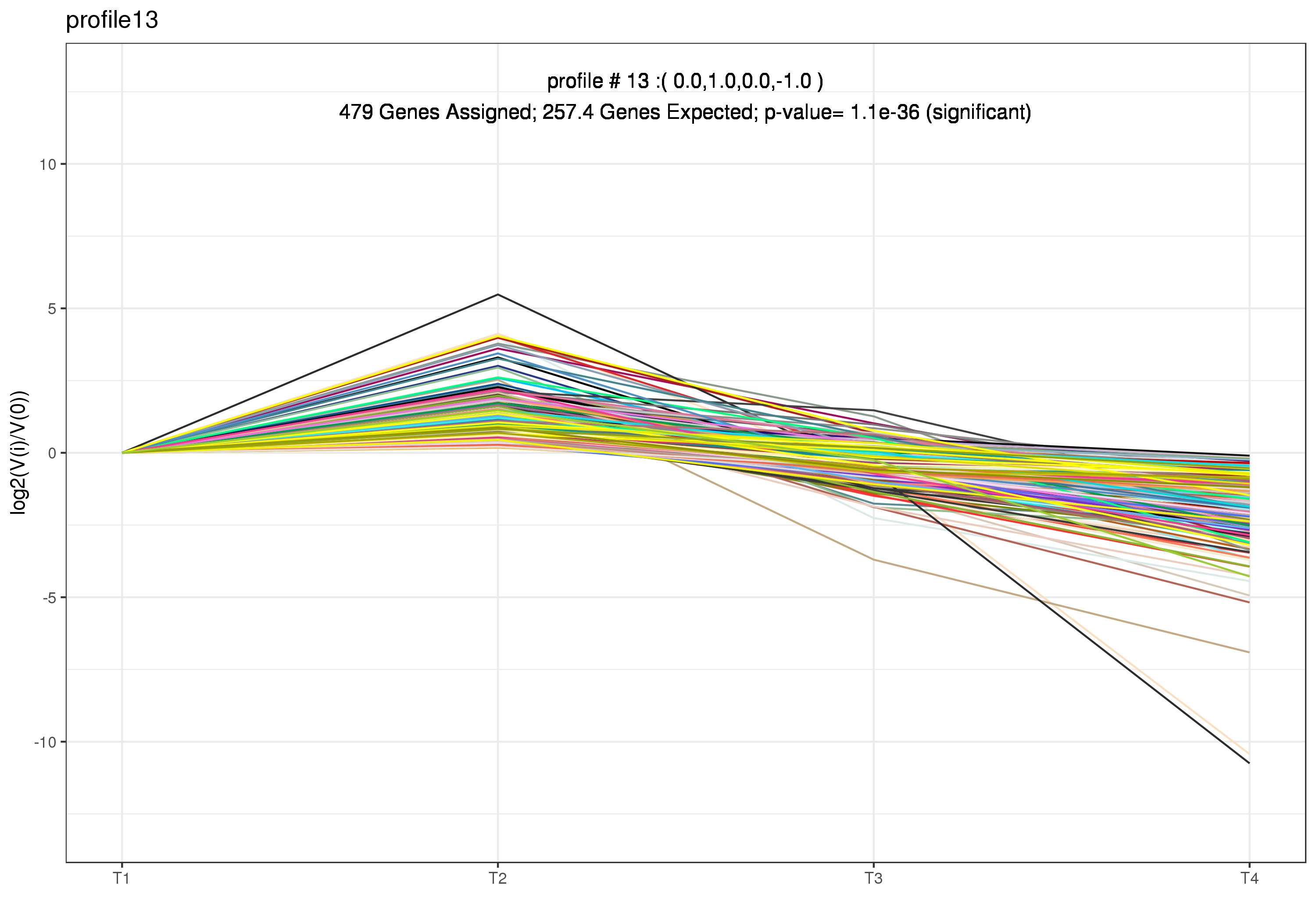 | Spliceosome  RNA transport  mRNA surveillance pathway  Peroxisome  Nucleotide excision repair | 31  14  7  4  3 | 4.71E-16  3.19E-04  9.79E-02  4.14E-01  4.32E-01 |
| profile 12  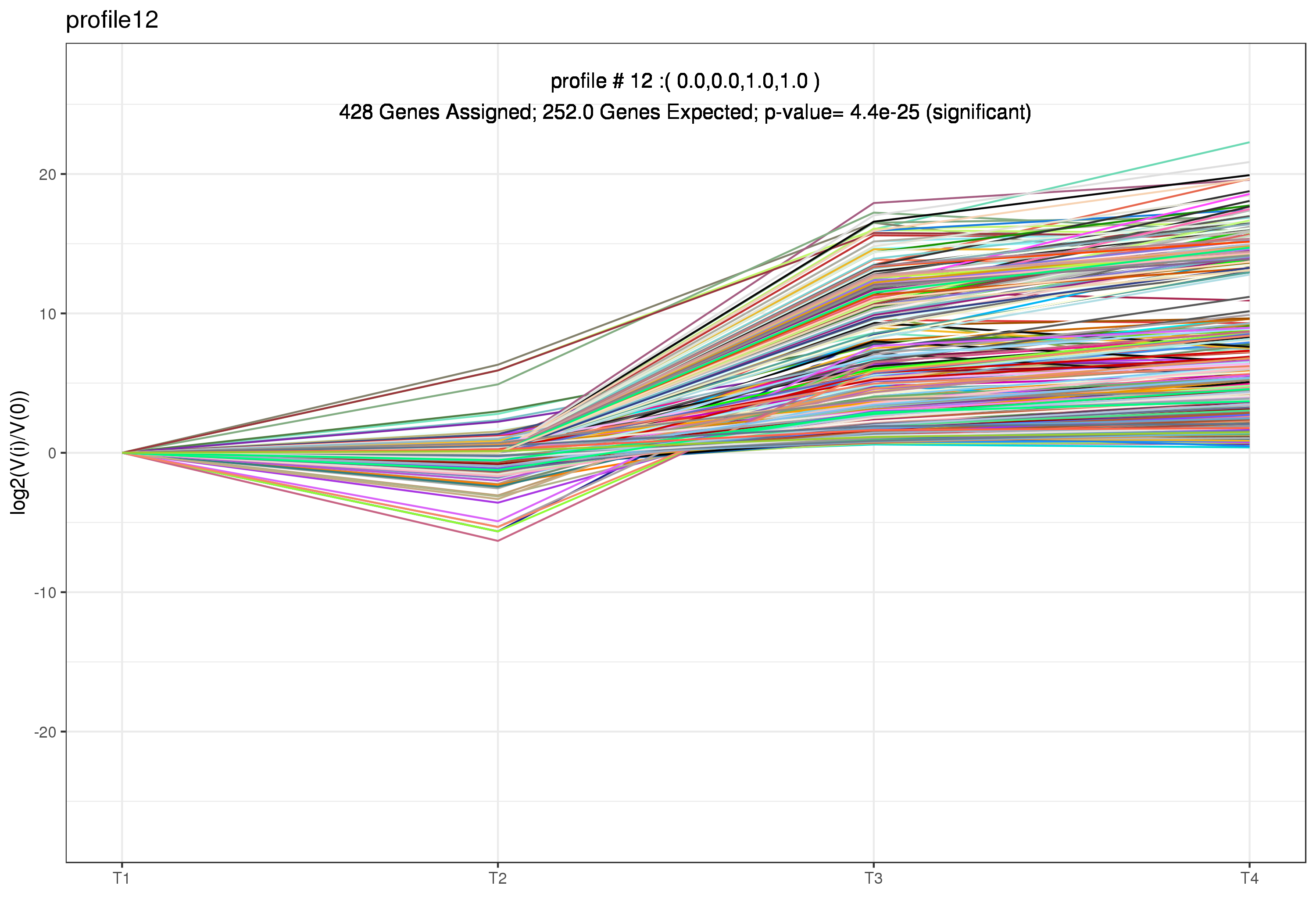 | Biosynthesis of secondary metabolites  Metabolic pathways  Starch and sucrose metabolism  Carbon metabolism  Phenylpropanoid biosynthesis | 45  63  11  17  9 | 0.000578938  0.00289582  0.006695631  0.034380117  0.067056404 |
| profile 7  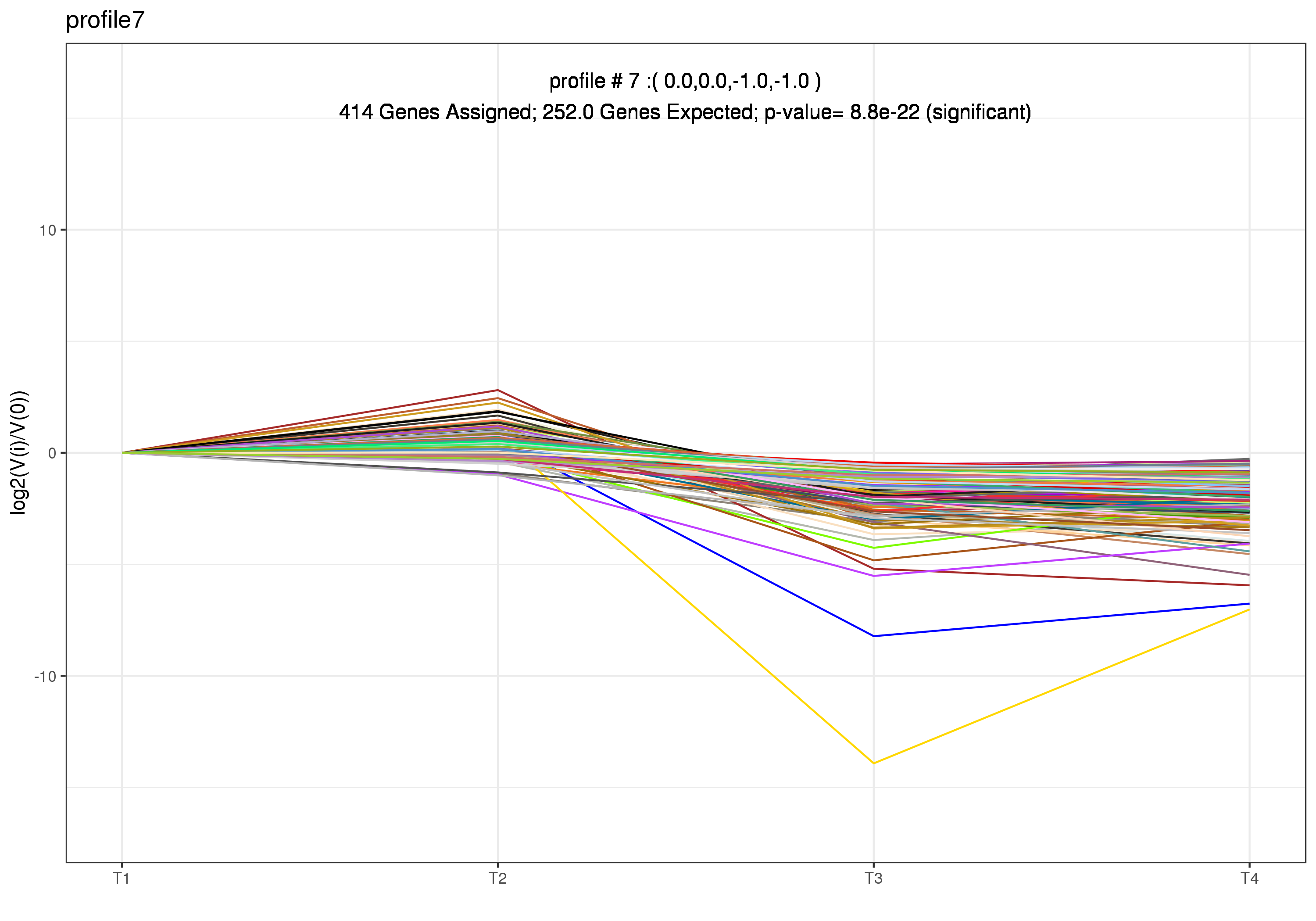 | mRNA surveillance pathway  Spliceosome  Sulfur metabolism  Ribosome biogenesis in eukaryotes  Isoflavonoid biosynthesis | 14  17  4  5  1 | 1.55E-06  7.15E-05  2.65E-02  2.72E-01  3.27E-01 |
| profile 9  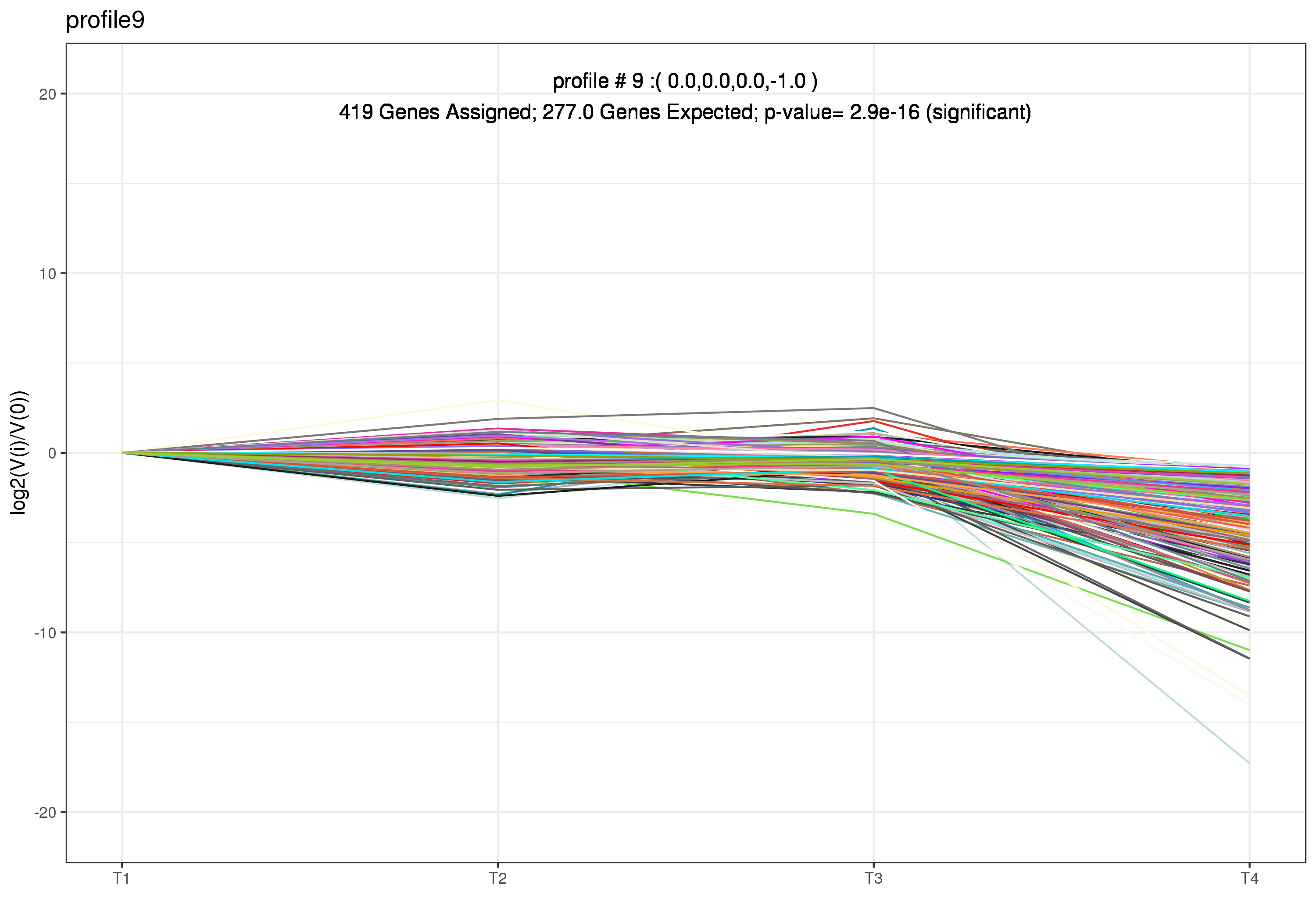 | Spliceosome  Ribosome biogenesis in eukaryotes  Protein processing in endoplasmic reticulum  Nitrogen metabolism  Carotenoid biosynthesis | 16  8  11  3  2 | 0.003822654  0.011194434  0.55853392  0.7619115  0.893585467 |
| profile 0  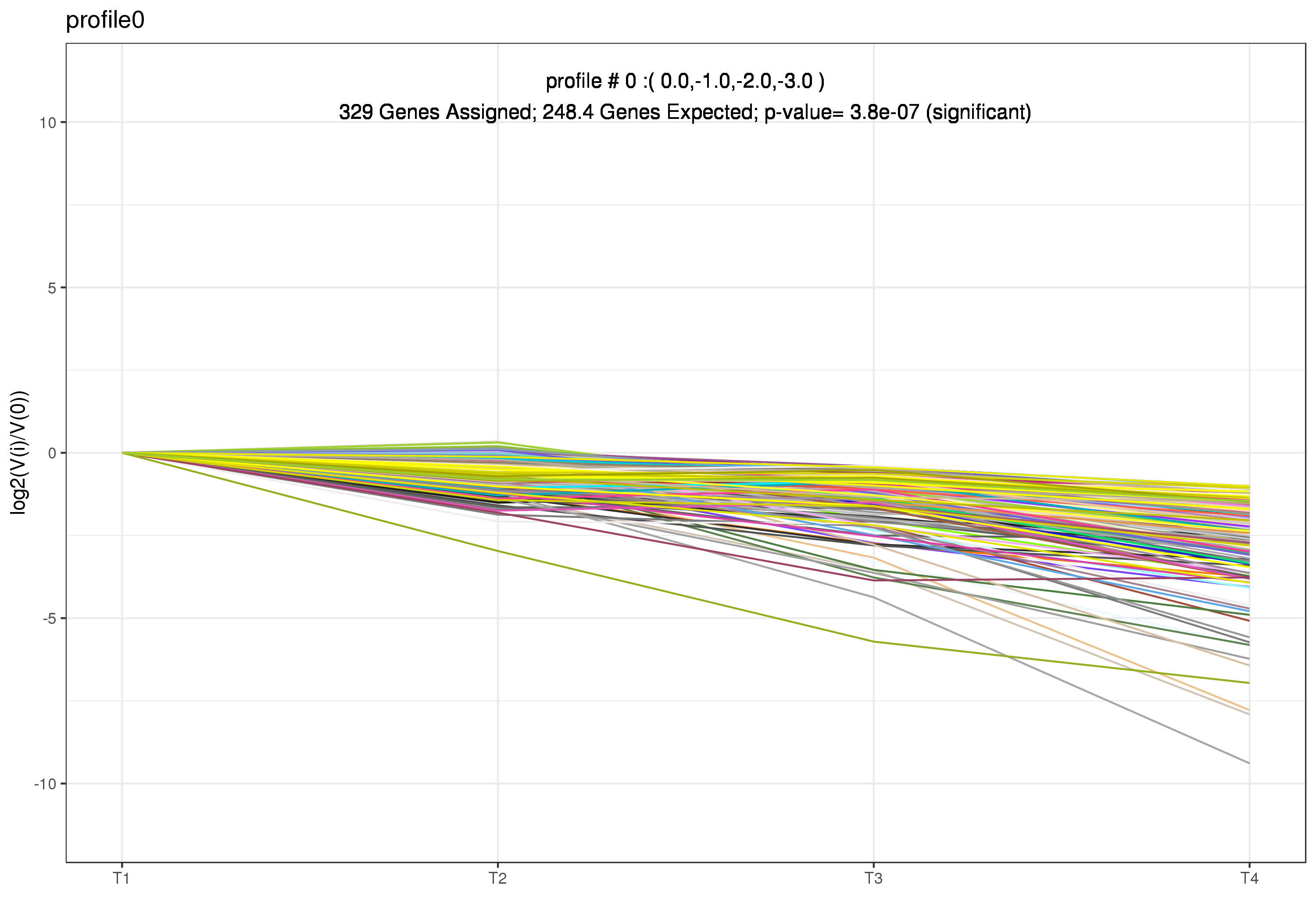 | Spliceosome  RNA polymerase  Pyrimidine metabolism  Stilbenoid, diarylheptanoid and gingerol biosynthesis  Nucleotide excision repair | 13  3  5  2  3 | 0.00738279  0.4062417  0.4062417  0.4062417  0.4062417 |
