# Supplementary material for: Plant hormonal changes and differential expression profiling reveal seed dormancy removal process in double dormant plant-herbaceous peony
Source: PLoS One. 2020 Apr 2;15(4):e0231117. doi: 10.1371/journal.pone.0231117 (PMC7117732; doi:10.1371/journal.pone.0231117)
Supplement: S5 Table — (DOC) [file pone.0231117.s005.doc]

**Table S5.** List of qRT-PCR primers used in this study

| Description | Gene_id | Forward(5’ to 3’) | Reverse(5’ to 3’) |
| --- | --- | --- | --- |
| Unigenes related to plant hormone metabolism and signal transduction | c66561_g1 | AGACTCTTTTCCCGTTACACCCT | ATTTTCCCTCTGAGAATCCATCC |
|  | c62105_g1 | AAAACCCTTGAACGCTGACATAG | ACAACTGCTTTACAAATCCCCAC |
|  | c53054_g1 | CTGCTCCGCCGTCATTCA | CTAACCGTTCCGTGCTATTCG |
|  | c72025_g1 | TTGACAACGAGAAGTTCACGGAG | ACGGCAAGTTCGGCGATAGT |
|  | c63749_g1 | TTCTCCAGTTACCAGTGGCTCC | TCACGCTTGACAGGTTTGTTTG |
|  | c69372_g1 | CGAACGCTGGGTTGGTCTACT | CAAACCGTCCGATGGTCTCC |
|  | c97332_g1 | TGCCGCTCGGTCAGGAAC | CCAAACTCGTCCGCAGCCT |
|  | c62439_g1 | AGCCTAGACGCCACCTGCTC | GCTTCCTGACCCTCCTTACTCC |
|  | c68709_g1 | TCCGCTGGACATGACCTTACC | CGGGGCTGACCTCTGAAAA |
|  | c56637_g1 | CTGTGAGGAAATCCACGACCC | GGCAATCCGCCGCTGTA |
|  | c57133_g1 | GCTTCAGAAACTGCAAGACGAGA | CACCAATGCTGGAACTACTACCCT |
|  | c33092_g1 | TTCTTTCGCCTCTGCTCCTTG | TCGCTTCCAGCCATTTTGC |
|  | c64904_g1 | CATACTAGCCAGCGACGGTTTC | CACTTCAGTTCCCCGAGCCT |
|  | c71286_g1 | CTCATCGTCTGCTTCATCCTCG | GCTACTTTCACCGTGTCCTCCA |
|  | c61546_g1 | GAATGTGACACCGTCTGCTCC | AAGTATAAGCCGATGACTCACCAA |
|  | c60942_g1 | AAATAGCGACTGGGTGGGTAAC | TAGCCAAAGCATCAAACAAAGTG |
|  | c57853_g1 | CTGCCCACCTCCGTCTGCT | TCCATAGGCTTTGACTCCTCGA |
|  | c65761_g1 | GGAGATGAAGCACGATAGCAACG | GCAGCAAAGATGACGCCAATG |
|  | c71204_g3 | TGCATATTTGTTTCCTCACCCC | TCCCTCCTCGTCACCCACTAC |
|  | c72165_g1 | CGGTCGGCAATTTTCTTTCAC | TGGCTATGGCTTCAATGTCAGG |
|  | c71028_g1 | ATGGTGTCGCTGTCATCACTATCA | GCGCCAGTAACTACTATTGCCTTC |
|  | c72798_g1 | GCGAGCAATGGGCTGGAG | CCGTTGGCATAATGGGGAAGT |
|  | c38112_g1 | CAGCCAAACCACTTCATCAACA | AACAGCCTTCTGGGGCAACT |
|  | c65334_g1 | AGATGGAGATTGGATGTTGGTGG | CATTTGGGAGGTGTCCTTGGAG |
|  | c60740_g1 | CAAACCCTCCCCAACAGCC | CAACGCCACCAGGAACCATA |
|  | c62979_g1 | GTCACCCAGTCGGTGAGCC | ATACCTGTTACCCGTGCCAAA |
| Unigenes selected randomly | c40652_g2 | ATGGCAGGAAATGAGGAGTGG | TGGTGTAAGGGCTGTAGGGTAAA |
|  | c67286_g1 | AAGCAAAATCGGCGAGAATAG | AGAGCGTCCATAGAGGTGAGGTA |
|  | c67048_g1 | GAAGGCTTGGAACAATGTATGG | TTCGCTGCTTTGGGAGTGTAT |
|  | c58056_g1 | CGAGAAGGAGCTTCCCACTG | CTACGCTTTGACGACCCAGAT |
|  | c48565_g1 | TTGGGGACGGGCTATGTCA | GTGGCGGCTTTGGATGTTT |
|  | c67058_g1 | TGCCCTACCAAGCCACATACA | TGCTGCCATTCCAGCTACATT |
|  | c62776_g1 | AATCCCCTTGTCCTGCTTCC | TGTTGAGTCTTTTGACGGTTCTG |
|  | c17645_g1 | GGGGTTTCAGCCAAAGCAA | CCCGACAGTTCCAACAGAGTG |
|  | c65021_g1 | CTGGGGCTCTGCTGGTCTT | CGTGGTCGCCGTAGTTTATG |
|  | c40383_g1 | GAGAAGTGTTCGCAGAGCAGC | CGTCACGATTTGGTATAGAGGG |
|  | c56669_g1 | TTTGCTATGGAAGTGAGGAAACG | TGGTGGTCGGAAAGTAACCC |
|  | c57809_g2 | GCCTCCTGCCCGTTGTATG | GCAAATTCGATCTTGAAGTCCCT |
|  | c62272_g1 | GTCACTGTTTGCCTTGGTGTTT | TTTGTTCCCTGGTGTTGCTG |
|  | c61415_g1 | CATCAAAATGGCACCCAAGA | TTCCACTGTCATCCAAGTTAGCA |
|  | c62757_g1 | TGCCACAGAAAATGAGGATGAT | TTGCTTGCTCTTGCCTTCG |
|  | c61871_g1 | AGGATCTTGTCGAACACCCAA | CTTCAGCAGAAACCCACCATTA |
|  | c52800_g1 | CATTTATCCCCAGGAACGAGC | ACCTTCTTAAACTGGCGGTCTC |
|  | c69674_g1 | AAGCCAGAAACTCAGGCATCC | GCCCATATTTCCTCCAAGACC |
|  | c61289_g1 | AAGAGGCTGAAATTCCATACGTC | CCCCTTGGTAGGCTTCGTTA |
|  | c52163_g1 | TGGAGAACACCAACCAGGAAC | ACCATGAATTTTGCGGTAAGC |
|  | c72419_g1 | CAATGGATCAATCCCAACGAG | TCCTGAAGTGTAGCAATGTCGC |
|  | c65238_g1 | TTCGACCACTAGATAGAGCCACA | CACAGATTTCAGCACCAACACC |
|  | c59761_g1 | TGAAAGGAGATTCTGTGAGACCG | CCTCCATGCAAAGTTGAAATGA |
|  | c55190_g1 | TCCAATGGCGTCTCGTTTTA | GTGTTGGATGAGCGAAGAGTGT |
|  | c68442_g2 | AGCTTTTCCTTGGCTACTTCACA | AAATTCTGCCGCTCCTTCC |
|  | c69857_g1 | CCACAGCAGCCATCCAAGG | CAACCCGATAAGCCGAGCA |
|  | c52536_g1 | ATCTCCCTCATCCATTTCTTCTG | TCCATCCCTTCTGCTTTTCATA |
|  | c74259_g1 | CTACCGAGCCCATATTTTATTCC | TGATTCTACGCCATTTGTCCTT |
| Reference gene | *PlActin* | GGTCTATTCTTGCTTCCCTC | CCCTCTGCGTCTACACTTTC |
