# Supplementary figures and images for: Plant hormonal changes and differential expression profiling reveal seed dormancy removal process in double dormant plant-herbaceous peony
Source: PLoS One. 2020 Apr 2;15(4):e0231117. doi: 10.1371/journal.pone.0231117 (PMC7117732; doi:10.1371/journal.pone.0231117)

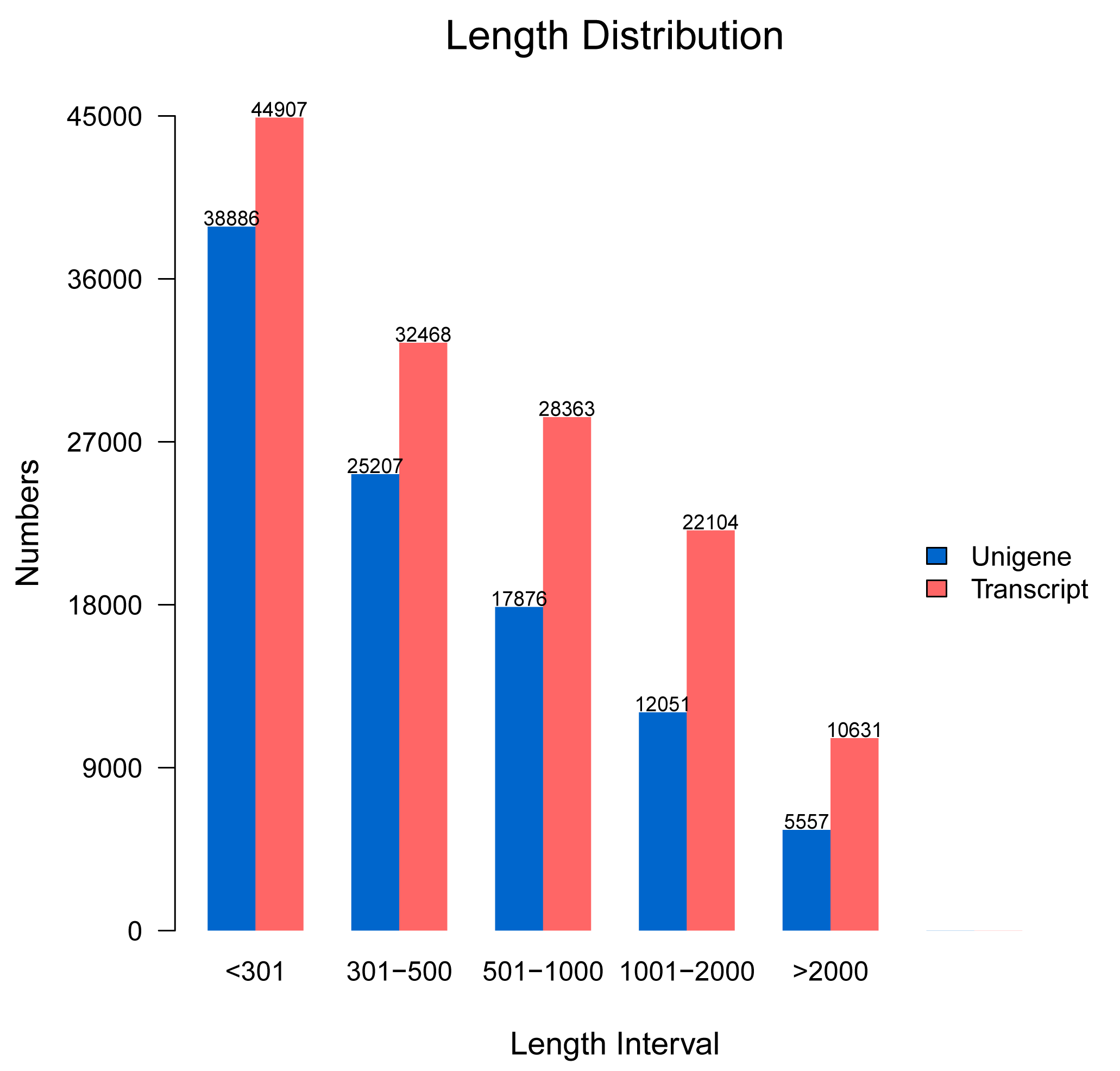

Supplement: S1 Fig — (TIF) [file pone.0231117.s006.tif]

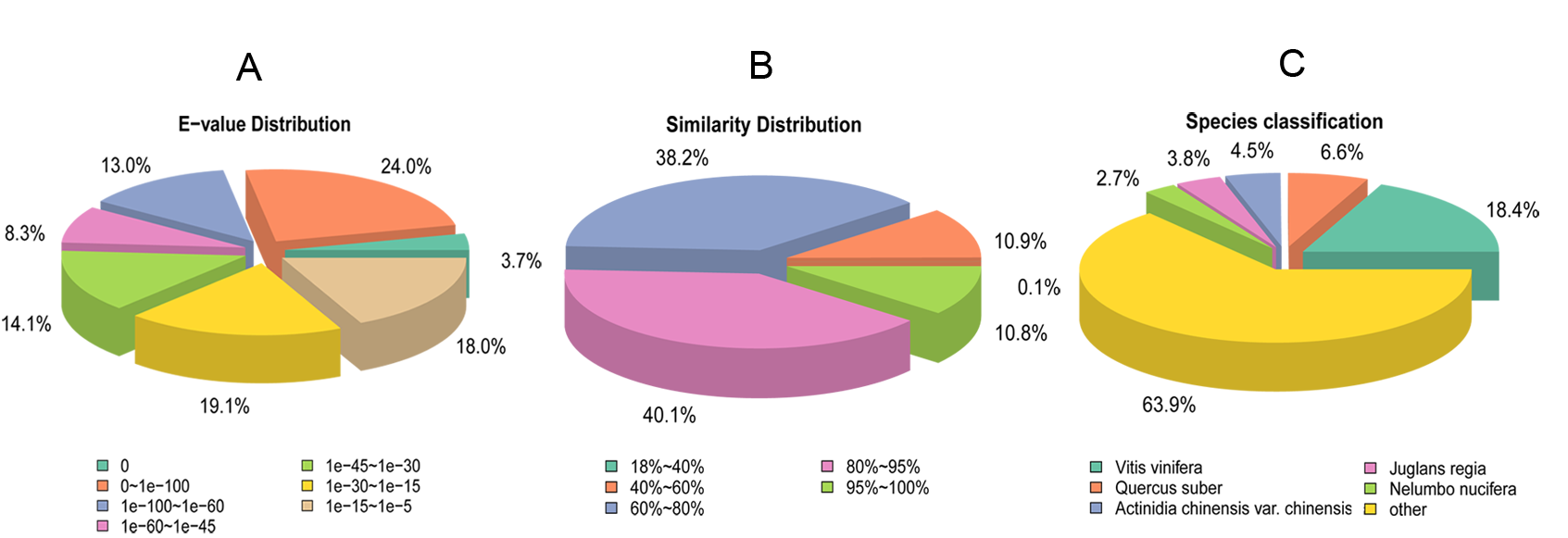

Supplement: S2 Fig — E-value distribution of the top BLAST hits for each unique sequence (A), Similarity distribution of the top BLAST hits for each unique sequence (B), Species distribution of the top BLAST hits for all homologous sequences (C). (TIF) [file pone.0231117.s007.tif]

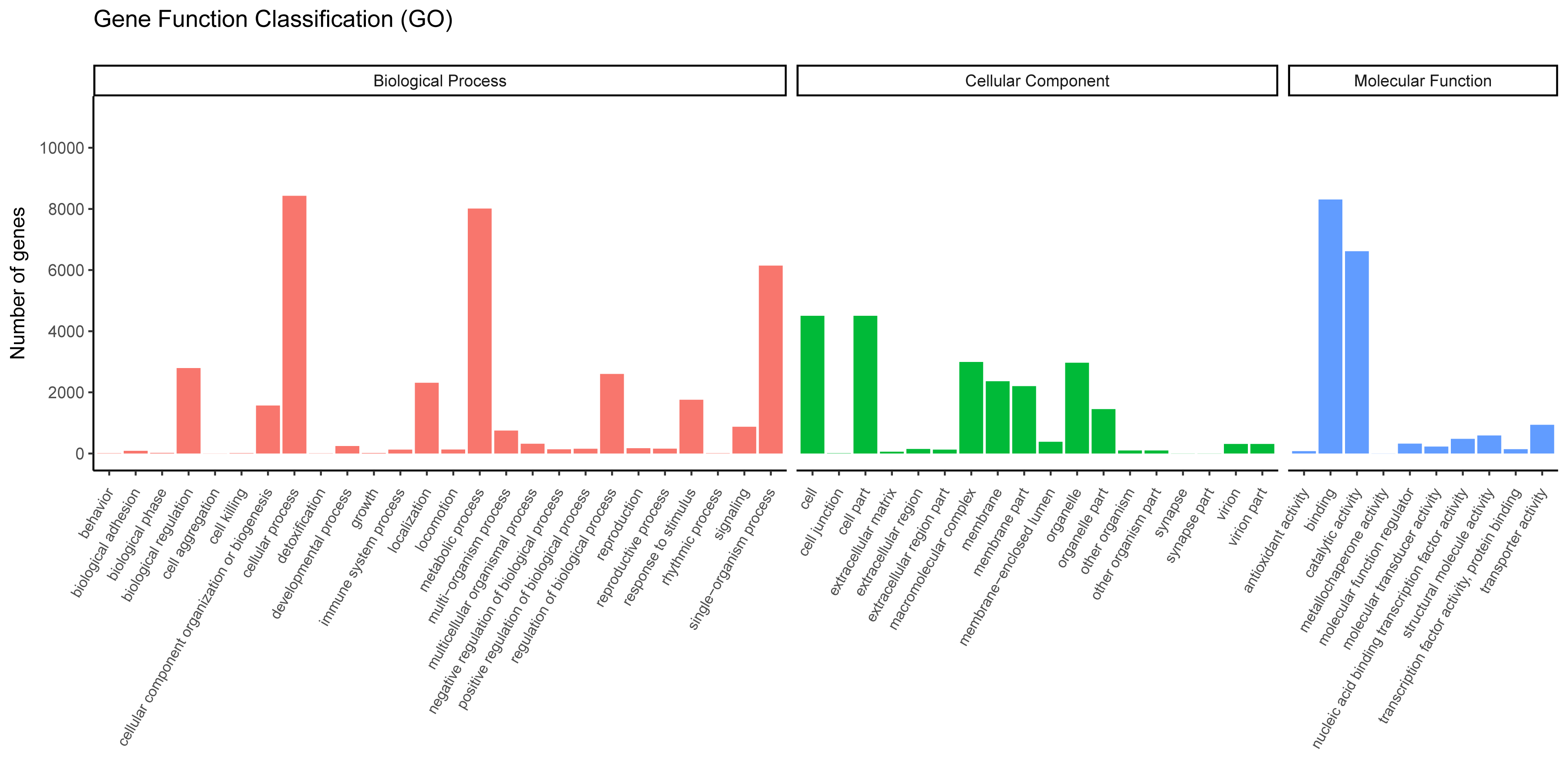

Supplement: S3 Fig — The 15,673 matched unigenes were classified into 3 functional categories: molecular function, biological process and cellular component. (TIF) [file pone.0231117.s008.tif]

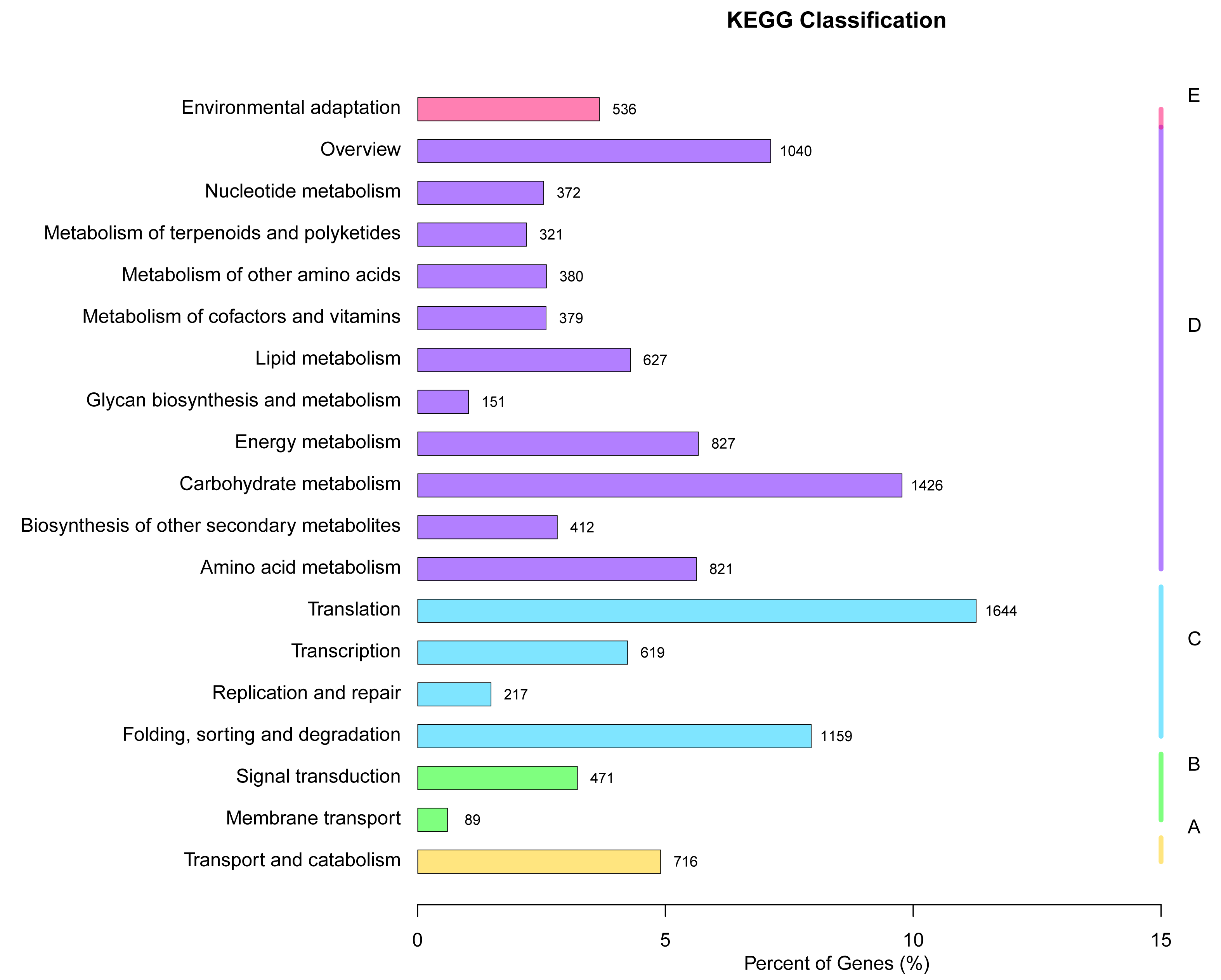

Supplement: S4 Fig — A total of 14,581 were assigned to 5 KEGG biochemical pathways: metabolism, genetic information processing, organism system, cellular processes and environmental information processing. (TIF) [file pone.0231117.s009.tif]

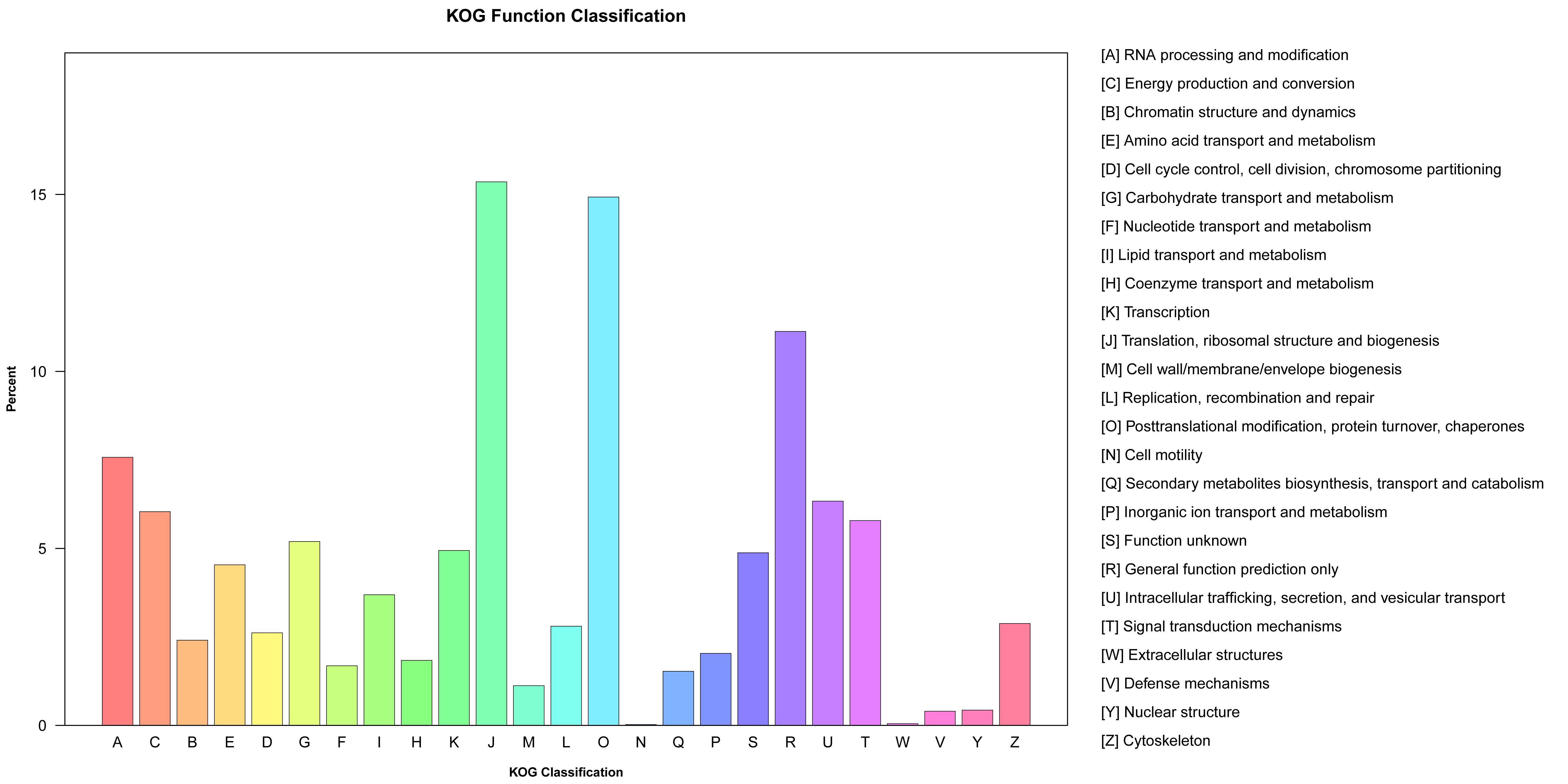

Supplement: S5 Fig — 9114 unigenes showed significant similarity to sequences in KOG databases, and were clustered into 26 categories. (TIF) [file pone.0231117.s010.tif]

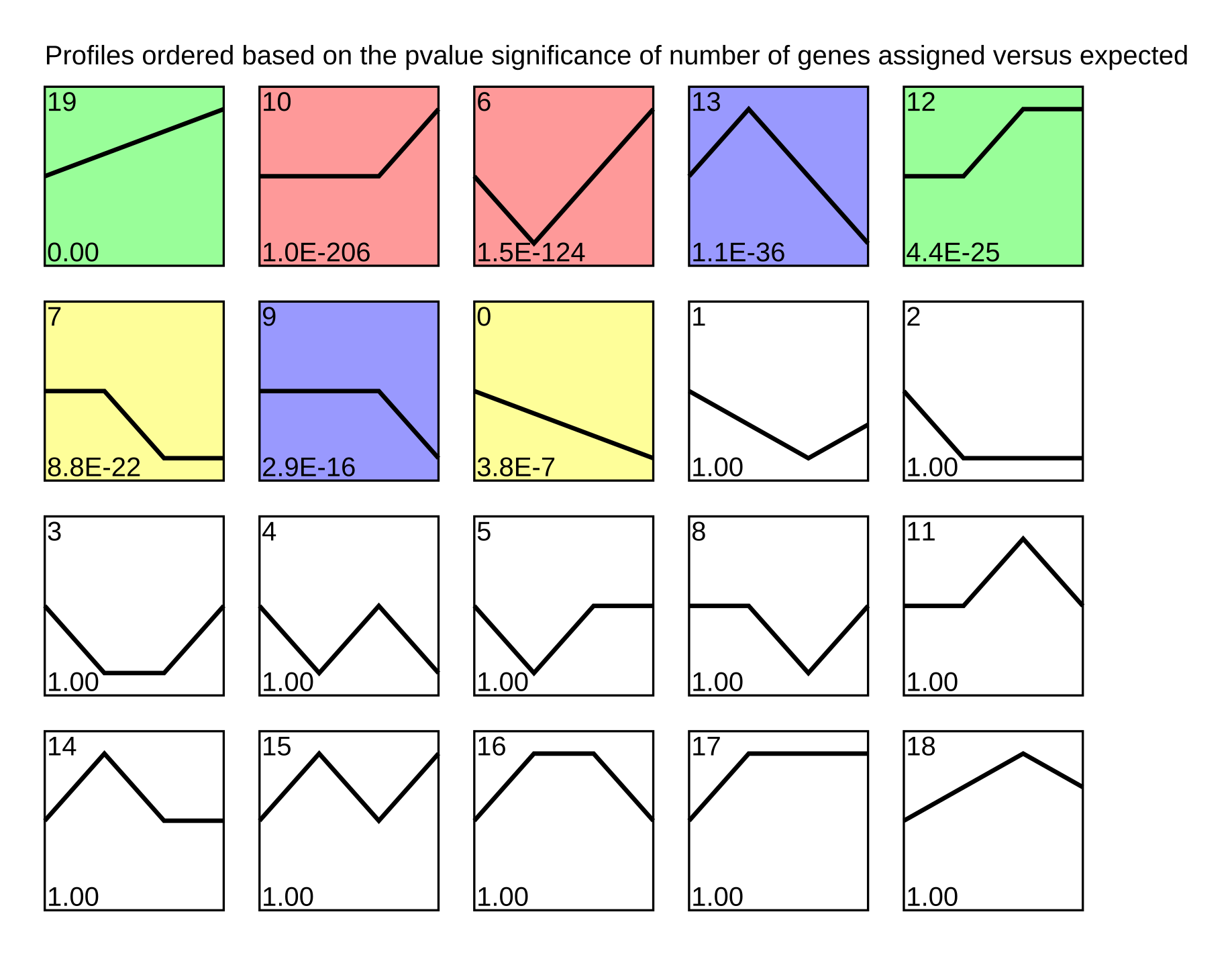

Supplement: S6 Fig — DEGs were clustered into 20 expression profiles. Profiles with P < 0.01 were separately subjected to KEGG pathway enrichment. (TIF) [file pone.0231117.s011.tif]

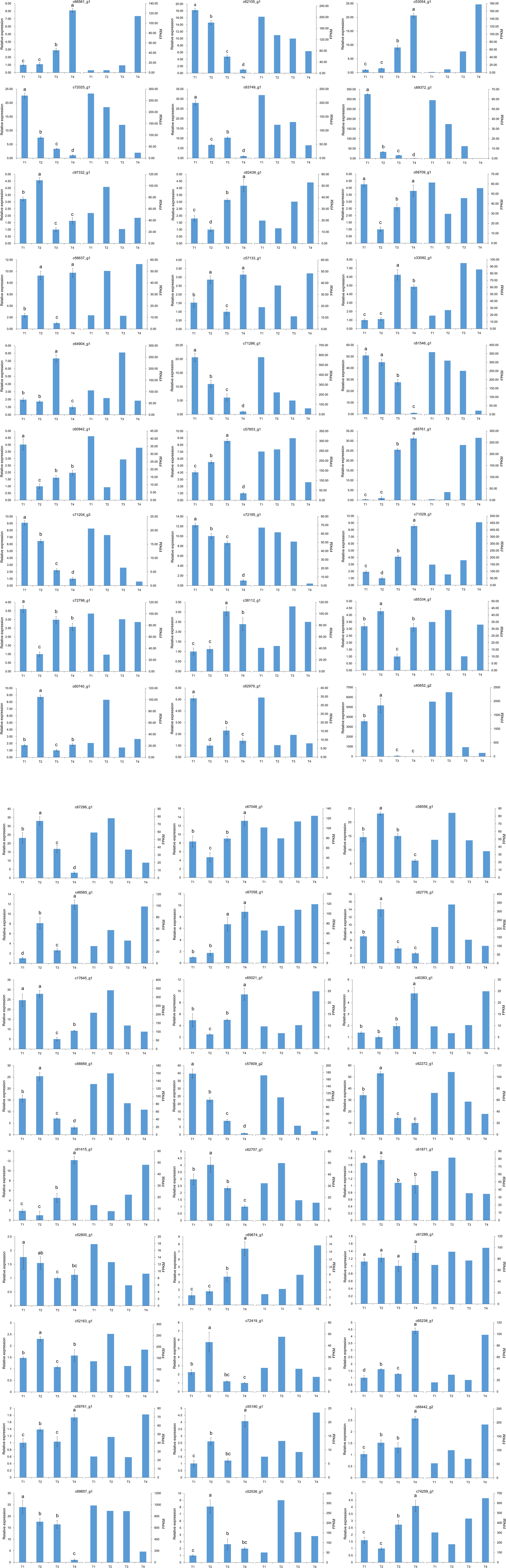

Supplement: S7 Fig — Data from qRT-PCR were presented as means with standard errors of three replications. Different lower case letter (a–d) indicates the significant difference among four stages at P < 0.05, dry seed (T1); imbibition seed (T2); the radicle breakthrough seed coat (T3); the germ break out (T4). (TIF) [file pone.0231117.s012.tif]

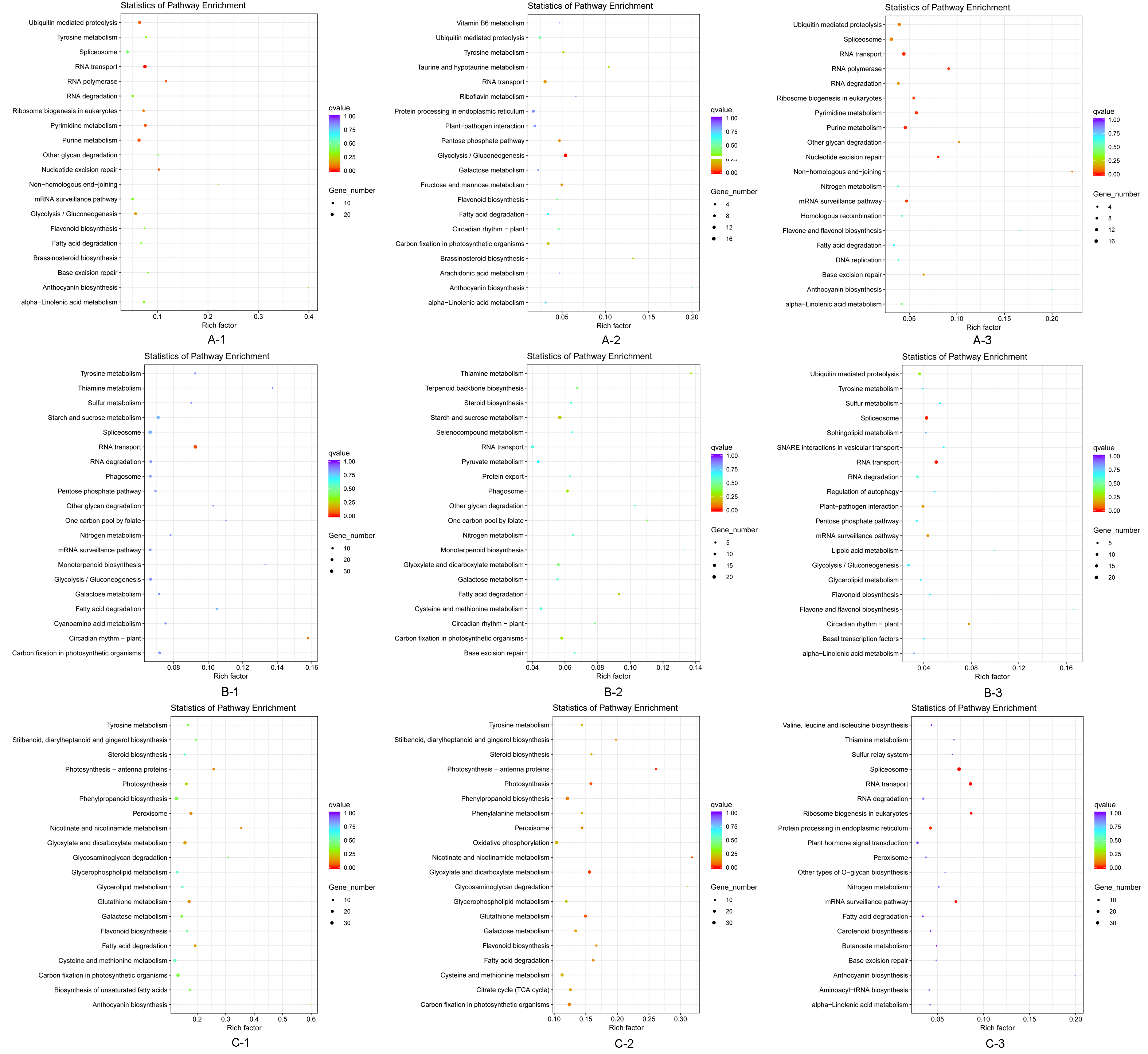

Supplement: S8 Fig — T2vsT1: total DEGs (A-1), up-regulated DEGs (A-2), down-regulated DEGs (A-3); T3vsT2: total DEGs (B-1), up-regulated DEGs (B-2), down-regulated DEGs (B-3); T4vsT3: total DEGs (C-1), up-regulated DEGs (C-2), down-regulated DEGs (C-3). (TIF) [file pone.0231117.s013.tif]
